# A Grapevine TTG2-Like WRKY Transcription Factor Is Involved in Regulating Vacuolar Transport and Flavonoid Biosynthesis

**DOI:** 10.3389/fpls.2016.01979

**Published:** 2017-01-05

**Authors:** Alessandra Amato, Erika Cavallini, Sara Zenoni, Laura Finezzo, Maura Begheldo, Benedetto Ruperti, Giovanni Battista Tornielli

**Affiliations:** ^1^Department of Biotechnology, University of Verona Verona, Italy; ^2^Department of Agriculture, Food, Natural Resources, Animals and Environment, University of Padova Padova, Italy

**Keywords:** flavonoids, grapevine, petunia, vacuolar acidification, WRKY

## Abstract

A small set of TTG2-like homolog proteins from different species belonging to the WRKY family of transcription factors were shown to share a similar mechanism of action and to control partially conserved biochemical/developmental processes in their native species. In particular, by activating P-ATPases residing on the tonoplast, PH3 from *Petunia hybrida* promotes vacuolar acidification in petal epidermal cells whereas TTG2 from *Arabidopsis thaliana* enables the accumulation of proanthocyanidins in the seed coat. In this work we functionally characterized *VvWRKY26* identified as the closest grapevine homolog of *PhPH3* and *AtTTG2*. When constitutively expressed in petunia *ph3* mutant, VvWRKY26 can fulfill the PH3 function in the regulation of vacuolar pH and restores the wild type pigmentation phenotype. By a global correlation analysis of gene expression and by transient over-expression in *Vitis vinifera*, we showed transcriptomic relationships of *VvWRKY26* with many genes related to vacuolar acidification and transport in grapevine. Moreover, our results indicate an involvement in flavonoid pathway possibly restricted to the control of proanthocyanidin biosynthesis that is consistent with its expression pattern in grape berry tissues. Overall, the results show that, in addition to regulative mechanisms and biological roles shared with TTG2-like orthologs, VvWRKY26 can play roles in fleshy fruit development that have not been previously reported in studies from dry fruit species. This study paves the way toward the comprehension of the regulatory network controlling vacuolar acidification and flavonoid accumulation mechanisms that contribute to the final berry quality traits in grapevine.

## Introduction

Grape berry needs a relatively long period to develop during which its tissues undergo several physiological and biochemical changes leading to the final composition and quality of ripe fruit. The primary and secondary metabolites mainly responsible for grape quality at ripening are compartmentalized in the vacuolar lumen where the physiological conditions favor their stabilization. During early berry development, both mesocarp and skin cells accumulate and store high concentrations of organic acids in their vacuoles, as malic and tartaric acids, which have remarkably low pH values (Ruffner, 1982). The acidification mechanism has been described in fruit of many species, including grapevine (Milner et al., 1995; Müller et al., 1996; Suzuki et al., 1999; Terrier et al., 2001). A combined proton pumping system constituted by V-PPases and V-ATPases influences the electrochemical potential gradient and allows the active transport of sugars, ions and organic acids across the tonoplast.

Another mechanism recently identified in *Petunia hybrida* for vacuole acidification requires *PhPH1* and *PhPH5* encoding two interacting P-ATPase transmembrane transporters located on the tonoplast. The functioning of both these pumps is essential for creating the proper acidic conditions in petal epidermal cells where anthocyanins are stored, whereas the expression of *PhPH5* alone is required for proanthocyanidin (PA) deposition in the seed coat (Verweij et al., 2008; Faraco et al., 2014). In Arabidopsis the *AtTT13*, homolog of *PhPH5*, functions as a proton pump in the tonoplast of seed coat endothelium cells and generates the driving force for the vacuolar uptake of PA precursors (Appelhagen et al., 2015). There is little information on the existence of a similar acidification mechanism in fleshy fruits. The lack of expression of the *PhPH5/AtTT13* citrus homolog was proposed as being responsible for the low acidity in the juice of Faris “sweet” lemon (*Citrus limon*) variety (Aprile et al., 2011). The homologs of both *PhPH1* and *PhPH5/AtTT13* were identified in grapevine and were shown to replace the function of the respective endogenous genes when expressed in petunia (Faraco et al., 2014; Li et al., 2016), suggesting the existence of a similar acidification mechanism in grapevine.

In petunia and Arabidopsis the expression of such P-ATPases is controlled by a MYB-bHLH-WDR (MBW) complex constituted by a MYB transcription factor (PhPH4 and AtMYB5/AtTT2, respectively), a bHLH transcription factor (PhAN1 and AtTT8, respectively) and a WD-regulatory protein (PhAN11 and AtTTG1, respectively) (Quattrocchio et al., 2006; Gonzalez et al., 2009; Li et al., 2009; Faraco et al., 2014).

The WRKY transcription factor PhPH3 has recently been characterized in petunia as an additional key regulator of vacuolar acidification, acting downstream and in concert with the MBW complex and controlling the expression of *PhPH1* and *PhPH5* (Verweij et al., 2016). The *ph3* mutants show an increase of petal homogenate pH that causes a shift in petal color, an abnormal seed development and female sterility (Verweij et al., 2016).

The functional homolog of PhPH3 in Arabidopsis is AtTTG2 that controls the seed PA and mucilage deposition, and the development of endothelium and trichomes (Johnson et al., 2002; Ishida et al., 2007). Similarly to PhPH3, AtTTG2 interacts with the MBW complex for the control of the vacuolar transport of PAs during seed coat development by regulating the expression of *AtTT13* and also by directly activating the expression of *TRANSPARENT TESTA 12* (*AtTT12*), a MATE transporter of glycosilated epicathechin PA precursors (Debeaujon et al., 2001; Gonzalez et al., 2016). Another functional homolog of AtTTG2 was identified in *Brassica napus*. BnTTG2 was shown to affect trichome development when over-expressed in *B. napus* plants and was capable of rescuing the trichome phenotype of Arabidopsis *TTG2* mutant (Li et al., 2015).

PhPH3, AtTTG2 and BnTTG2 are all members of the plant-specific WRKY family whose regulatory functions have mainly been associated with the response to abiotic and biotic stresses but also with various developmental and physiological processes (Rushton et al., 2010; Schluttenhofer and Yuan, 2015). These transcription factors have a 60 amino acidic sequence, called WRKY domain, containing the conserved sequence WRKYGQK and a zinc-finger motif, and is responsible for recognition of W-boxes in the promoters of target genes (Ciolkowski et al., 2008). The WRKY proteins are classified in groups I–III based on both the number of WRKY domains and the features of their zinc-finger motif (Eulgem et al., 2000). PhPH3, AtTTG2 and BnTTG2 proteins are all members of group Ia with two WRKY domains and a C2H2 zinc-finger motif (Johnson et al., 2002; Xie et al., 2005; Li et al., 2015; Verweij et al., 2016).

In *Vitis vinifera* the WRKY family is composed of 59 members (Wang M. et al., 2014) and the few proteins functionally characterized so far seem to be mostly involved in plant response to biotic or abiotic stress (Marchive et al., 2007, 2013; Li et al., 2010; Liu et al., 2011; Zhu et al., 2012; Jiang et al., 2015; Merz et al., 2015). In this study, we characterized VvWRKY26, the closest grapevine homolog to PhPH3 and AtTTG2. We showed that VvWRKY26 is able to complement the petunia *ph3* mutation. Moreover, from detailed analyses of expression in grapevine organs and the study of *VvWRKY26* transiently over-expressing grapevine plantlets we provided evidence for a role of VvWRKY26 in the regulation of vacuolar transport and flavonoid accumulation in grape berry.

## Materials and methods

### Bioinformatics

To identify the grapevine WRKY sequence homolog to PhPH3, the predicted proteome of the near-homozygous PN40024 genotype of *Vitis vinifera* “Pinot noir” 12X V1 version (http://genomes.cribi.unipd.it) was queried using the conserved amino acid sequence of PhPH3 (Verweij et al., 2016).

VvWRKY26 protein was aligned against the full amino acid sequences of AtTTG2 (AT2G37260), BnTTG2 (AJD07412) and PhPH3 (AMR43368) using Muscle.

The phylogenetic tree was constructed from a Muscle alignment of a portion of the protein sequences that includes the C-terminal WRKY domains up to the stop codon. The final phylogenetic tree was obtained using the neighbor-joining method and bootstrap analysis (1000 replicates) and Mega6 software (Tamura et al., 2013). The following GenBank accession numbers were used: AtWRKY2 (AT5G56270), AtWRKY4 (AT1G13960), AtWRKY6 (AT1G62300), AtWRKY7 (AT4G24240), AtWRKY18 (AT4G31800), AtWRKY23 (AT2G47260), AtWRKY35 (AT2G34830), AtWRKY36 (AT1G69810), AtWRKY42 (AT4G04450), AtWRKY62 (AT5G01900), AtWRKY70 (AT3G56400), AtTTG2 (AT2G37260), VpWRKY1 (ACY69975), VpWRKY2 (ADD70008), VpWRKY3 (AEN71143), VvWRKY01 (AAT46067), VtWRKY11 (B2G283), VvWRKY33 (AHG99400), MrWRKY30 (Jiang et al., 2015), GhWRKY3 (ADI52618.1), PcWRKY1 (AAD55974.1), PcWRKY3 (AAC49528), PcWRKY4 (AAG35658), OsWRKY6 (DAA05071), OsWRKY76 (DAA05141), WIZZ (BAA87058.1), CaWRKY1 (AAO86686), CjWRKY1 (BAF41990).

The online MEME Suite (http://meme-suite.org/doc/overview.html; Bailey et al., 2009) was used to discover protein motifs with an expected value lower than 2 × 10^−12^ using the following search parameters: four to twelve residues (minimum to maximum length), any number of repetitions, and 10 motifs maximum.

### Plant material and growth conditions

All petunia plants used in this study derived from the Amsterdam University collection. Complementation analysis of *VvWRKY26* was conducted out in a line mutated in *PH3* locus that is heterozygous for the allele *ph3*^*B*2267*FP*^ harboring 7 bp footprint left by the excision of the transposon *dTpH1* and for the allele *ph3*^*R*49^ featuring the deletion of part of the coding sequence (Verweij et al., 2016). The *ph3* mutant, the wild type R27 and the transgenic lines were cultivated under normal glasshouse conditions. For the transient over-expression of *VvWRKY26* in grapevine, plantlets of *Vitis vinifera* cv. Sultana were micropropagated *in vitro* and cultivated in a growth chamber at 25°C with a 16-h photoperiod.

### Gene cloning and genetic transformation

The *VvWRKY26* cDNA sequence was amplified by PCR from grapevine post fruit set seed cDNA of cv. Corvina using Pfu DNA polymerase (Promega) and the primers listed in Supplementary Table 1. The generated PCR fragment was purified and directionally cloned into the Gateway entry vector pENTR/D-TOPO (Invitrogen) reflecting the presence of a 5′-CACC-3′ leader sequence in each forward primer. The product was verified by sequencing and transferred into the binary over-expression vector pK7GW2,0 (Laboratory of Plant Systems Biology, PSB; Ghent University, Belgium; https://gateway.psb.ugent.be/vector/show/pK7WG2/search/index/overexpression/any) by site-specific LR recombination.

For stable transformation of *P. hybrida*, the *35S:VvWRKY26* construct was inserted into *Agrobacterium tumefaciens* strain EHA105 by electroporation. *P. hybrida* plants were transformed using the leaf disc method (van der Meer, 1999) and regenerated transgenic shoots were transferred to soil and hardened off in a temperature-controlled glasshouse.

For transient transformation of *Vitis vinifera* cv. Sultana, the *35S:VvWRKY26* construct was transferred to *Agrobacterium tumefaciens* strain C58C1 by electroporation. As control, Agrobacterium was also transformed with a pK7WG2.0 vector containing a non-coding sequence. Six *in vitro* 5 weeks old plants of grapevine cv. Sultana were immersed in each bacterial suspension and vacuum infiltrated (2 × 2 min at 90 kPa). After agroinfiltration, plantlets were rinsed with sterile water and allowed to recover *in vitro* for 6 days before collecting material for RNA extraction and transcriptomic analysis.

### Measurement of petunia petal extract pH

Petal limb tissues collected from 10 distinct flowers, representing 10 biological replicates, were collected from the *ph3* mutant, the wild type R27 and each transgenic line. The pH of petal extracts was measured by grinding the petal limbs in 6 mL of distilled water, as described by Quattrocchio et al. (2006). The actual pH values measured for each line showed some variation over time, possibly reflecting the variable environmental conditions in the glasshouse, but the differences between transgenic and control lines were constant. The absolute pH values could therefore reliably be compared between samples measured in the same experiment.

### Anthocyanin determination in petunia petals

Petal limb tissues collected from three distinct flowers, representing three biological replicates, were collected from the *ph3* mutant, the wild type R27 and each transgenic line. Powdered petal samples were extracted in 8 volumes (w/v) of methanol acidified with 0.1% (v/v) hydrochloric acid in an ultrasonic bath at 40 kHz for 15 min at room temperature. Total amount of anthocyanins was determined by spectrophotometer at λ = 540 nm using malvidin 3-glucoside as standard.

### RNA extraction and expression analyses by qPCR

For molecular characterization of petunia plants, total RNA was isolated from flowers using TRIzol® Reagent (Invitrogen) following the manufacturer's instructions. For molecular characterization of grapevine cv. Sultana plantlets, total RNA was isolated from 20 to 40 mg of young and well expanded ground leaves using Spectrum™ Plant Total RNA kit (Sigma-Aldrich) according to the manufacturer's instructions.

All RNA samples were quantified with the NanoDrop 2000 instrument (Thermo Scientific Technologies) and 1 μg aliquots were treated with DNase I (Promega) and then reverse transcribed using Super-Script™ III Reverse Transcriptase (Invitrogen) according to the manufacturer's instructions.

The expression profiles analyzed in petunia petals and grapevine organs were determined by qPCR as described by Zenoni et al. (2011), using the SYBR Green PCR master mix (Applied Biosystems) and a Mx3000P real time PCR system (Stratagene). Each expression value, relative to an actin internal control in petunia experiments and *VvUBIQUITIN1* in grapevine, was determined using three biological triplicates. In all qPCR analyses, gene expression data (cycle threshold values) were used to quantify relative gene expression by using the efficiency corrected method described in Pfaffl (2001). The primer sequences used for qPCR analysis are listed in Supplementary Table 1.

### Microarray analyses

For microarray analysis RNA quality and integrity were determined using Bioanalyzer Chip RNA 7500 series II (Agilent).

For microarray analysis on petunia petals the cDNA synthesis, labeling, hybridization and washing reaction were done according to the NimbleGen Arrays User's Guide (V 3.2). Hybridization was performed on a NimbleGen microarray 111012_PAX_PT_expr_HX12 chip (Roche, NimbleGen Inc.), representing 27,627 predicted genes on the basis of the *Petunia axillaris* genome. The microarray was scanned using a ScanArray 4000XL (Perkin-Elmer) at 532 nm (Cy-3 absorption peak) and GenePix Pro7 software (Molecular Devices) according to the manufacturer's instructions. Images were analyzed using NimbleScan v2.5 software (Roche), which produces Pair Files containing the raw signal intensity data for each probe and Calls Files with normalized expression data derived from the average of the intensities of the probes for each gene. The normalized gene expression data were finally converted in log_2_ values to process the data. A Pearson's Correlation analysis was conducted to evaluate the robustness of the three biological replicates in each sample. A gene was considered to be expressed if the normalized expression value was higher than the value obtained by averaging the fluorescence of a negative control present on the chip, for at least two or three biological replicates. A Significance Analysis of Microarrays (SAM) was implemented using TMeV software (http://mev.tm4.org).

For microarray analysis on Sultana leaves transiently over-expressing *VvWRKY26*, the cDNA synthesis, labeling, hybridization and washing reactions were performed according to the Agilent Microarray-Based Gene Expression Analysis Guide (V 6.5). A new Agilent custom microarray was designed on the 4-pack 44K format (Agilent Sure Print HD 4X44K 60-mer G2514F-048771). This custom microarray was created by using an Agilent's web-based application able to create custom microarray designs and oligolibraries (https://earray.chem.agilent.com/earray/), as described by Dal Santo et al. (2016). Scanning and Feature Extraction was performed by using an Agilent Scanner following the settings and parameters indicated in the instruction manual. After the extraction was completed successfully, the QC report for each extraction was analyzed to assess the quality of the overall hybridization procedure. A data-matrix was prepared selecting from each single sub-array outcome file the *gProcessedSignalvalues*, which are the raw fluorescence intensities of each probe. The data were normalized on the 75th percentile and a correlation analysis was then conducted to assess the consistency of the biological triplicates. Correlation matrixes were prepared using R software and Pearson's Correlation Coefficient (PCC) as the statistical metric. Data reported in the normalized data-matrix were used to determine the genes differently expressed in different samples by performing a *T*-test (TMeV).

### Correlation analysis of expression

Correlation analysis of expression was performed exploiting the grapevine co-expression database VTCdb (http://vtcdb.adelaide.edu.au/Home.aspx) using the *VIT_08s0040g03070* relative to *VvWRKY26* and selecting HRR co-expression measure (Wong et al., 2013).

### Berry fixation, embedding, and *in situ* hybridization (ISH)

Berries of *Vitis vinifera* cv. Corvina clone 48 were collected from grapevines grown in a commercial vineyard at Montorio (45° 27′ 17″ North, 11° 03′ 14″ East, Verona, Italy) in the 2010/2011 growing season. The samples were collected at 8 weeks before véraison (corresponding to fruit set stage) and at 1 week after véraison. After harvest, the samples were fixed with 2% formaldehyde and 0.25% glutaraldehyde in PBS buffer (pH 7.5) under vacuum overnight.

Fixed berries were rinsed five times in PBS and then dehydrated in four consecutive solutions containing increasing ethanol concentrations (25, 50, 75, and 100%). Post fixation steps were performed with four overnight treatments in ethanol solutions containing increasing xylene concentrations (25, 50, 75, and 100%). The embedding was performed progressively substituting the xylene with Paraplast Plus (Thermo Fisher Scientific). Tissue sections (7 μm thick) were cut with a 2035 Leica microtome (Leica Microsystems GmbH, Wetzlar, Germany), floated on warm water, and immobilized on poly-L-lysine coated slides to aid handling. The slides were then air-dried at 37°C and used for ISH. All the following ISH steps were done using an adaptation of a universal liquid handling robot, Freedom EVO 100, from Tecan and performed as described by Begheldo et al. (2013).

The antisense and sense *VvWRKY26* probes 749 bp in length were selected by PCR on leaf cDNA of *Vitis vinifera* cv. Corvina with the primer sets listed in Supplementary Table 1.

## Results

### VvWRKY26 phylogenetic analysis

A previous search for *WRKY* genes in the genome of the near-homozygous PN40024 genotype of *Vitis vinifera* cv. Pinot noir (Jaillon et al., 2007) led to the identification of 59 sequences belonging to this family (Wang M. et al., 2014). Among these, sequence *VIT_08S0040G03070* named *VvWRKY26*, located on chromosome 8, was identified as the closest gene to *AtTTG2* by Wang M. et al. (2014). In this study, we performed a Blast search in the grapevine PN40024 12X V1 genome showing that *VvWRKY26* is also the closest grapevine gene to *PhPH3*, as recently shown by Verweij et al. (2016).

The full-length coding region of *VvWRKY26* was amplified from cv. Corvina post fruit set seed cDNA, cloned and submitted to GenBank under the accession number KX823961.

*VvWRKY26* contains a 1434 bp open reading frame encoding a protein of 477 amino acidic residues with a predicted mass of 52.27 kDa and a calculated pI = 8.84. The deduced amino acid sequence is 99% similar to the predicted one of cv. Pinot noir with only one different amino acid at position 324 (Supplementary Figure 1).

**Figure 1:**
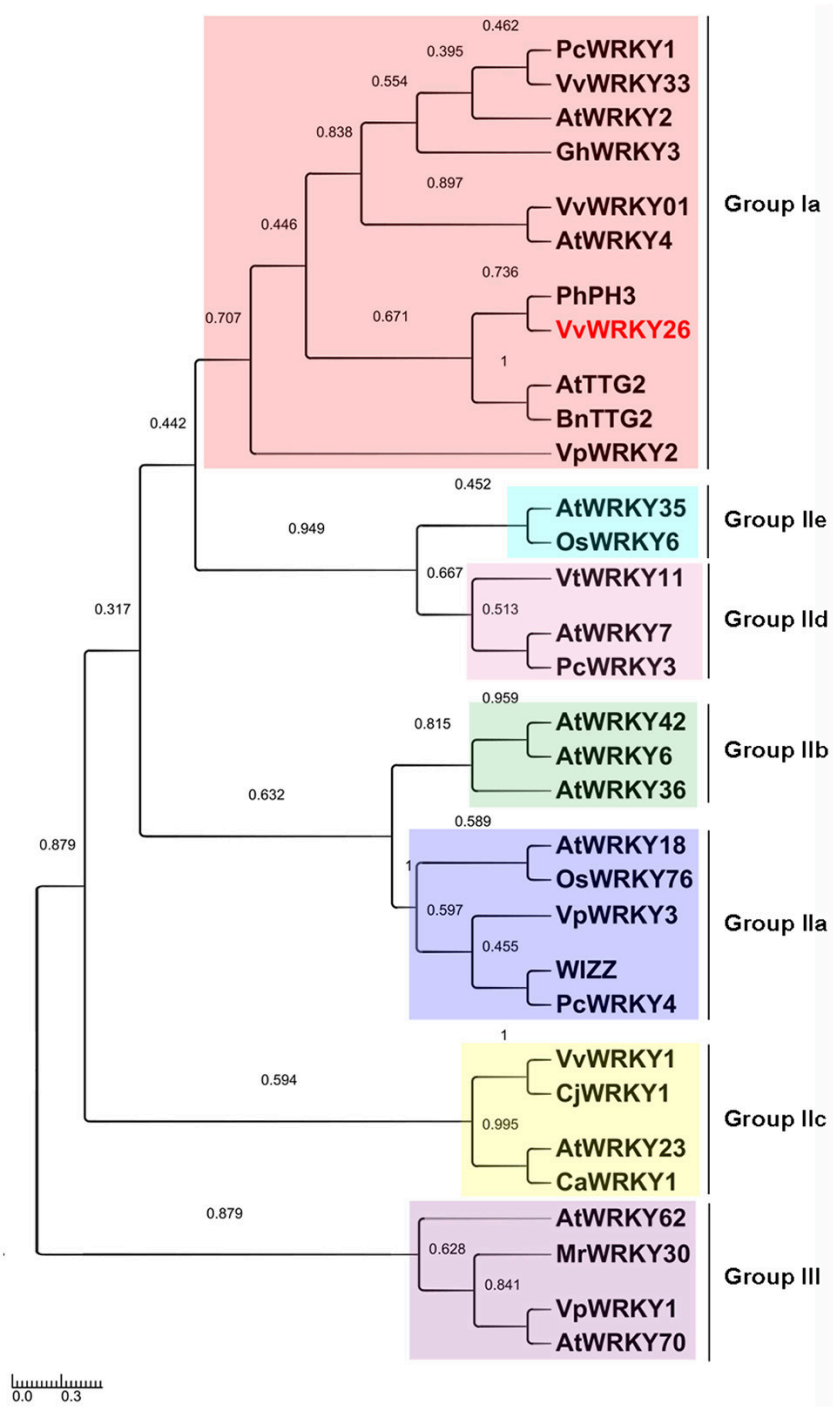
**Phylogenetic tree including WRKYs belonging to different species**. The analysis, based on alignments of the C-terminal WRKY domain, was performed using the neighbor-joining method by the Mega version 6 program (Tamura et al., 2013). The scale bar represents the number of substitutions per site and the numbers next to the nodes are bootstrap values from 1000 replicates. The accession numbers are reported in the Materials and methods section.

A phylogenetic analysis of 32 WRKY proteins, including characterized WRKY TFs of grapevine and of different plant species, showed that VvWRKY26 belongs to the cluster of Group I WRKYs as PhPH3, AtTTG2 and BnTTG2, as also shown by Verweij et al. (2016) (Figure 1). The cluster contains other grapevine WRKY factors that are mainly involved in response to biotic and abiotic stresses, such as VvWRKY33 and VpWRKY2 (Li et al., 2010; Merz et al., 2015) and VvWRKY01 (VvWRKY2) related to lignin synthesis (Guillaumie et al., 2010). In the same cluster are AtWRKY2 and AtWRKY4 that play roles in seed germination, in pollen development (Jiang and Yu, 2009; Guan et al., 2014) and in sugar metabolism (Hammargren et al., 2008), respectively, and PcWRKY1 and GhWRKY3 involved in the response against plant pathogens (Eulgem et al., 1999; Turck et al., 2004; Guo et al., 2011). The other grapevine WRKY sequences included in this phylogenetic analysis all belong to different clusters and groups, further underlying functional differences with VvWRKY26.

**Figure 2:**
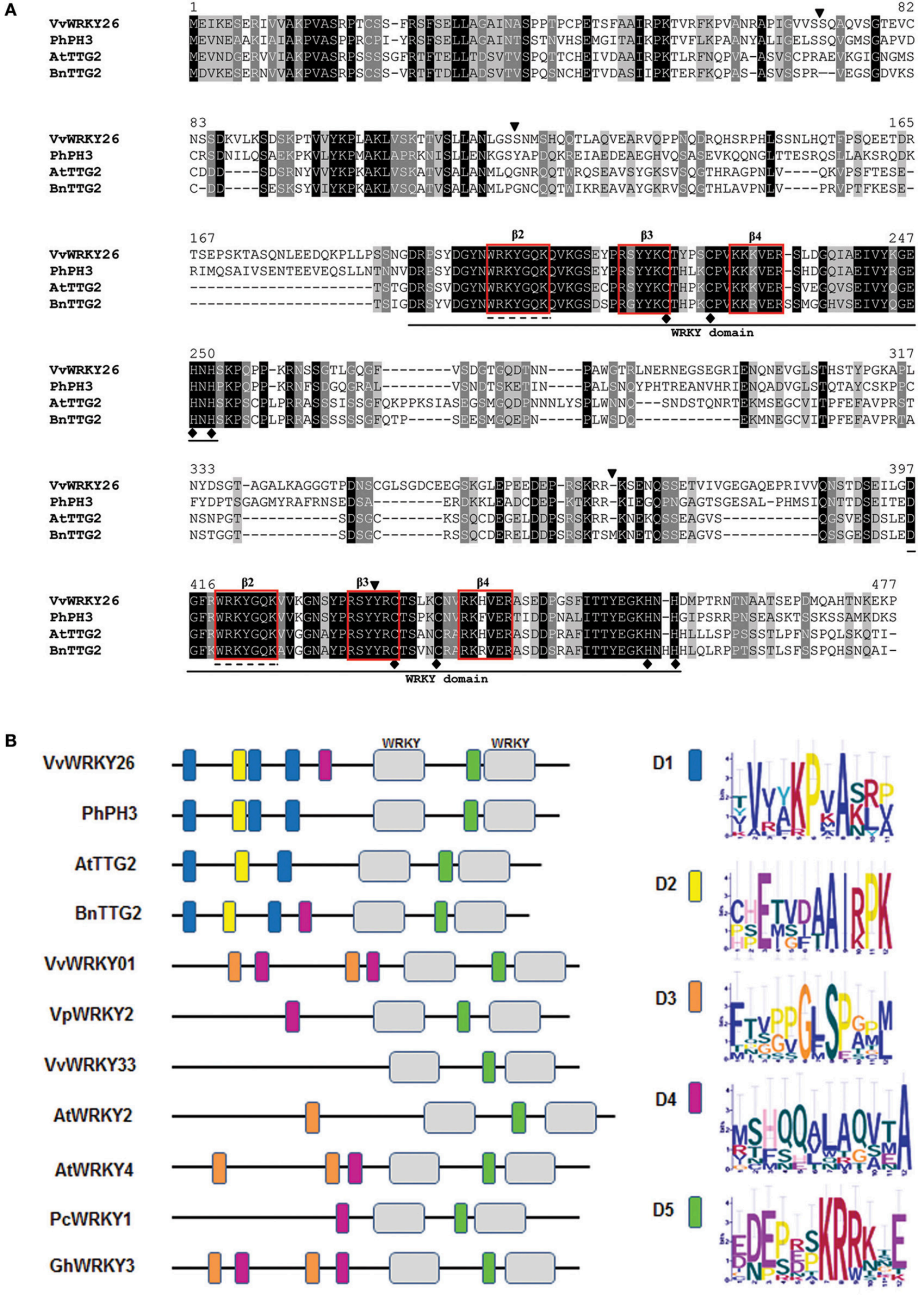
**Analyses of primary protein structures. (A)** Alignment of VvWRKY26, PhPh3, AtTTG2, and BnTTG2 predicted sequences. Identical, conserved and similar residues are shown in black, light gray and dark gray, respectively. The black lines below the alignment locate the two WRKY domains, with the conserved sequences WRKYGQK and the C2H2 zinc-finger motifs underlined by dashed lines and black dots, respectively. Triangles above the alignment indicate the position of the introns. The first three introns are conserved in all three species, while the last one close to the 3′ UTR is present only in petunia and grapevine. Red squares individuate β2, β3, and β4 in the WRKY domains. **(B)** Protein domain organization of Group I WRKY factors represented by colored boxes identified by MEME Suite. The consensus sequence of the motifs is reported.

*VvWRKY26* is characterized by the presence of four introns and shares the position of the first three with the homologs *PhPH3, AtTTG2* and *BnTTG2* (Figure 2A; Johnson et al., 2002; Li et al., 2015; Verweij et al., 2016). Only for *VvWRKY26* and *PhPH3* is there an additional intron in the region corresponding to the C-terminal WRKY domain (Verweij et al., 2016).

The alignment of the amino acidic sequences revealed that, among all, VvWRKY26 is closer to PhPH3 with 54% sequence similarity compared with 48 and 46% to AtTTG2 and BnTTG2, respectively (Figure 2A). Analysis of the protein primary structure revealed the presence of two WRKY domains in correspondence to amino acids 200 and 401, classifying VvWRKY26 as a group Ia member of the WRKY family, as PhPH3, AtTTG2, and BnTTG2 (Figures 1, 2A; Xie et al., 2005; Verweij et al., 2016). In all these four WRKY proteins both WRKY domains are highly conserved and contain the amino acid sequence **WRKYGQK** at the N-terminal ends together with the C2H2-type zinc-finger motifs **C–X**_4_**–C–X**_22_**–H–X**_1_**–H** (Johnson et al., 2002; Li et al., 2015; Verweij et al., 2016). As proposed for AtWRKY1 using a crystal structural model (Brand et al., 2013), three strands β2, β3, and β4 that are necessary for the specific protein-DNA interaction can be identified within the WRKY domains of the four proteins (Figure 2A).

By further analysing the Group I WRKY factors from Figure 1 by MEME bioinformatic tool, we highlighted other conserved protein motifs (Figure 2B). Among these, the D1 and D2 domains located at the N-terminus are specific of the TTG2-like WRKY proteins (Figure 2B; Supplementary Figure 2). Moreover, the domain D5 containing a putative nuclear localization signal **KRRK**x**E** is common to all sequences and was previously identified also by Wang M. et al. (2014)

Overall, our analyses allowed the identification of VvWRKY26 as the grapevine homolog of both petunia PH3 and Arabidopsis TTG2, and highlighted unique domains shared by TTG2-like WRKYs.

### Heterologous expression of *VvWRKY26* in petunia *ph3* mutant

**Figure 3:**
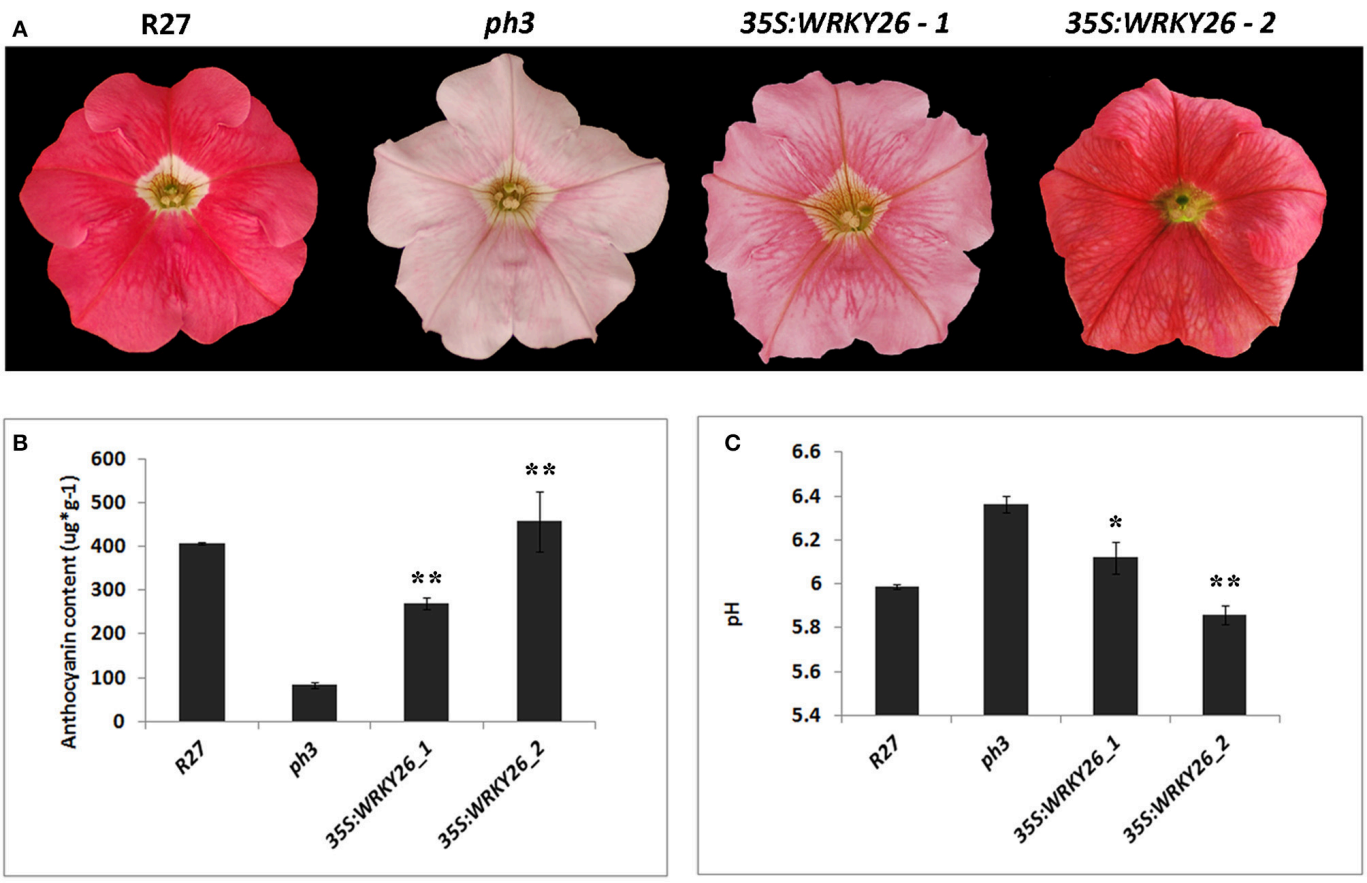
**Complementation analysis in ***ph3*** petunia mutant with ***35S:VvWRKY26***. (A)** Phenotype of untransformed flowers of the wild type R27 and mutant *ph3* lines compared to transgenic flowers of two lines expressing *VvWRKY26*. **(B)** Total anthocyanin content (μg^*^g^−1^ fresh weight) of petal limb extracts from untransformed R27 and *ph3* lines and transgenic plants determined by spectrophotometry at 540 nm. Purified malvidin 3-glucoside was used as a standard. Data represent the mean of three biological replicates ± SE. Asterisks indicate significant difference against the *ph3* mutant line (^**^P > 0.01). **(C)** The pH values of crude petal limb extracts from untransformed R27 and *ph3* lines and transgenic plants. Each pH value is the mean of 10 biological replicates ± SE. Asterisks indicate significant difference against the *ph3* mutant line (^*^P > 0.05; ^**^P > 0.01).

To gain information on the role of VvWRKY26 and ascertain its ability to fulfill the function of *PhPH3* we performed complementation analysis in a *P. hybrida ph3* mutant. The coding sequence of *VvWRKY26* was expressed under the control of the constitutive *35S* promoter in the mutated petunia line. The *ph3* mutation affects the vacuolar acidification in petal epidermal cells, resulting in a higher pH and a shift of the anthocyanin absorption spectrum (Verweij et al., 2016). Therefore, the corollas of the *ph3* mutant line present a pale pink pigmentation in comparison to the strong red color of the wild type line R27 (Figure 3A).

The transformation yielded 6 PCR positive plants with a range of petal color phenotypes, e.g. from evenly red-pigmented corollas similar to the R27 wild type line, to pale pink like the mutant *ph3* flowers (Figure 3A). For further phenotypic characterization, we selected two independent lines (lines 4 and 2) showing intermediate (strong pink) and fully complemented (red) petal phenotypes, respectively, corresponding to low and high expression of *35S:VvWRKY26* as determined by qPCR (Figure 3A; Supplementary Figure 3). We also observed a strong pigmentation on the petal veins in both transgenic lines that was independent of the complementation level (Figure 3A). No other phenotypic effects were observed on transgenic petunia plants in comparison with the *ph3* mutant line.

The pH analysis of petal crude extracts revealed that both transgenic lines presented significantly lower pH compared to the *ph3* mutant and that only the fully red complemented line 2 presented pH values similar to the wild type R27 (Figure 3B), suggesting that VvWRKY26 fully restored the *ph3* mutation fulfilling the function of the endogenous PH3 in the regulation of vacuolar acidification.

We analyzed the anthocyanin content of petal extracts for each line. As shown in Figure 3C, surprisingly the *ph3* mutant was affected in the anthocyanin synthesis, accumulating significantly less anthocyanin content than the wild type R27 line. On the contrary, both transgenic lines expressing *VvWRKY26* presented anthocyanin levels comparable to the wild type R27 line, in a consistent manner with the levels of transgene expression (Figure 3C) suggesting that VvWRKY26 can also contribute to the control of anthocyanin synthesis.

In petunia the mutation in *PH3* gene causes seed abortion and female sterility indicating a role as yet uncharacterized in seed development (Verweij et al., 2016). In our complementation analysis, we observed that the expression of *VvWRKY26* partially overcame the sterility of the *ph3* line, in fact the self-fertilization of *VvWRKY26* over-expressing petunias yielded few capsules with seeds showing wild type phenotype (data not shown). However, because of the sterility of *ph3* mutant plants, the screening and comparison of the seed phenotypes of the self-pollinated transgenic and mutant plants was impossible.

### Transcriptomic analysis of petunia petals expressing *VvWRKY26*

To identify potential target genes of VvWRKY26, we compared the petal transcriptomes of *35S:VvWRKY26* plants and untransformed *ph3* mutant. Three independent transgenic lines showing fully complemented petal phenotype and comparable expression level of the transgene were chosen as biological replicates (lines 1, 2, and 5; Supplementary Figure 3). Total RNA was extracted from petals of each sample and hybridized against a NimbleGen microarray 111012_PAX_PT_expr_HX12 chip containing 27,627 probes representing the *Petunia axillaris* transcriptome (Zenoni et al., 2011). We identified 546 genes differentially expressed by implementing a Significance Analysis of Microarrays (SAM) and using a False Discovery Rate (FDR) of 0.1% (Microarray Gene Expression Omnibus database accession GSE86890). All transcripts were annotated using the functional information content of the *Petunia axillaris* transcriptome (Supplementary File 1; Zenoni et al., 2011). The most relevant differentially expressed genes were selected by considering the subset of up-regulated genes in each transgenic line with a fold change (FC) value ≥ 2 compared with mutant petals. Putative functional annotations were manually improved by BLAST analysis and those with no similarity to known sequences (no hit) were removed from the subset (Supplementary File 1).

The ectopic expression of *VvWRKY26* affects genes belonging to a wide range of cellular processes (Supplementary File 1). In particular, we observed the modulation of many genes putatively encoding transmembrane transporters (Table 1), such as an uncharacterized ammonium transporter (*PETAX069902_Contig1*) and the potassium channels *KAT2* (*PETAX007908_Contig1*) and *HAK5* (*PETAX015455_Contig1*) involved in stomatal opening and in the response to low K^+^ concentration, respectively (Sharma et al., 2013; Nieves-Cordones et al., 2014). Electron carriers and three lipoxygenases putatively involved in vacuolar trafficking and homeostasis were also up-regulated by the ectopic expression of *VvWRKY26* (Table 1). Interestingly, a transcript corresponding to a nodulin *N21-like* (*PETAX090339_Contig* 1) gene was one of the most induced genes with an FC of 49.78. The function of this class of genes is not yet understood, but the presence of several transmembrane domains in the proteins and structural homologies with bacterial multidrug exporters suggest a role in transport (Guillaumie et al., 2008).

**Table 1:** **Subset of up-regulated genes in ***ph3/35S:VvWRKY26*** petunia petals involved in secondary metabolism and in vacuolar trafficking and homeostasis**.

**ID_Code**	**Description**	**FC^a^**	
PETAX090339_Contig1	Nodulin Mtn21-like protein *(Populus trichocarpa)*	49.78	vacuolar trafficking and homeostasis
PETAX010001_Contig2	*predicted:* cytochrome P450 77A3-like *(Vitis vinifera)*	40.09	
PETAX010955_Contig1	PDR6 gene for ATPase coupled to transmembrane movement of substances *(Arabidopsis thaliana)*	6.66	
PETAX016561_Contig1	Lipoxygenase *(Nicotiana attenuata)*	5.70	
PETAX069902_Contig1	Ammonium transmembrane transporter *(Arabidopsis thaliana)*	5.30	
PETAX093411_Contig1	SKU5 similar gene for copper ion binding / oxidoreductase *(Arabidopsis thaliana)*	4.20	
PETAX008575_Contig1	Transcription factor/ zinc ion binding *(Arabidopsis thaliana)*	4.13	
PETAX043560_Contig1	GDSL-motif lipase *(Arabidopsis thaliana)*	3.94	
PETAX004540_Contig2	Lipoxygenase *(Arabidopsis thaliana)*	3.81	
PETAX019816_Contig1	SKU5 similar gene for copper ion binding / oxidoreductase *(Arabidopsis thaliana)*	3.57	
PETAX049570_Contig1	SKU5 similar gene for copper ion binding / oxidoreductase *(Arabidopsis thaliana)*	3.49	
PETAX017332_Contig2	Urea transmembrane transporter/ water channel *(Arabidopsis thaliana)*	3.49	
PETAX004540_Contig1	Lipoxygenase *(Arabidopsis thaliana)*	3.48	
PETAX007908_Contig1	KAT2 gene for potassium channel *(Arabidopsis thaliana)*	3.44	
PETAX002530_Contig1	Copper ion binding / electron carrier *(Arabidopsis thaliana)*	3.28	
PETAX027806_Contig1	Serine carboxypeptidase-like 25 *(Arabidopsis thaliana)*	3.05	
PETAX029839_Contig1	GLIP5 gene for carboxylesterase/ lipase *(Arabidopsis thaliana)*	3.05	
PETAX015455_Contig1	HAK5 gene for potassium ion transmembrane transporter *(Arabidopsis thaliana)*	3.03	
PETAX005065_Contig1	Pentatricopeptide repeat-containing protein, putative *(Ricinus communis)*	3.03	
PETAX028339_Contig1	GLIP4 gene for carboxylesterase/ lipase *(Arabidopsis thaliana)*	2.79	
PETAX016028_Contig1	RAV transcription factor (regulator of the ATPase of the vacuolar membrane) *(Arabidopsis thaliana)*	2.75	
PETAX009077_Contig1	SKU5 similar gene for copper ion binding / oxidoreductase *(Arabidopsis thaliana)*	2.74	
PETAX045174_Contig1	Carboxylesterase *(Arabidopsis thaliana)*	2.49	
PETAX059202_Contig1	DIR1 (defective in induced resistance)/lipid transporter *(Arabidopsis thaliana)*	2.41	
PETAX060333_Contig1	Extracellular Ca2 sensing receptor *(Nicotiana tabacum)*	2.38	
PETAX058844_Contig1	Cytochrome P450 *(Solanum lycopersicum)*	2.36	
PETAX031181_Contig1	Phospholipid transporter *(Arabidopsis thaliana)*	2.26	
PETAX054602_Contig1	Hf2 gene for flavonoid 3′, 5′-hydroxylase (F3′5′H) *(Petunia integrifolia)*	82.72	secondary metabolic processes
PETAX037083_Contig1	N-acetyl-glutamate synthase *(Solanum lycopersicum)*	21.43	
PETAX027750_Contig1	TT7 gene for flavonoid 3′-monooxygenase (F3′H) *(Arabidopsis thaliana)*	6.52	
PETAX005218_Contig1	UDP-glucosyl transferase *(Arabidopsis thaliana)*	5.01	
PETAX000309_Contig10	UDP-glucosyl transferase *(Arabidopsis thaliana)*	4.60	
PETAX000309_Contig38	Flavonol 4′-sulfotransferase *(Ricinus communis)*	4.11	
PETAX000309_Contig83	p-Coumarate 3-hydroxylase (C3H) *(Arabidopsis thaliana)*	3.39	
PETAX000309_Contig126	C-4 methylsterol oxidase *(Arabidopsis thaliana;* predicted *Vitis Vinifera)*	2.84	
PETAX000309_Contig127	C-5 sterol desaturase *(Arabidopsis thaliana)*	2.82	
PETAX000309_Contig145	Resveratrol/hydroxycinnamic acid O-glucosyltransferase *(Vitis labrusca)*	2.72	
PETAX000309_Contig169	UDP-glucosyl transferase *(Ricinus communis)*	2.49	
PETAX000309_Contig194	C-4 methylsterol oxidase *(Arabidopsis thaliana)*	2.29	
PETAX000309_Contig225	C-4 methylsterol oxidase *(Arabidopsis thaliana)*	2.12	
PETAX000309_Contig241	4-Coumarate-CoA ligase (4CL) *(Arabidopsis thaliana)*	2.08	
PETAX000309_Contig246	Flavonoid glucoyltransferase UGT73E2 (*Antirrhinum majus*)	2.04	
PETAX000309_Contig248	Putative UDP-glucuronosyltransferase *(Arabidopsis thaliana)*	2.03	
PETAX000309_Contig258	UDP-glucosyl transferase *(Arabidopsis thaliana)*	2.00	

a *FC, fold change*.

Related to the flavonoid pathway, the microarray analysis revealed a strong induction of the Petunia *HF2* gene encoding for the *FLAVONOID 3*′*, 5*′*-HYDROXYLASE (F3*′*5*′*H, PETAX054602_Contig1)* and, to a lesser extent, of the *AtTT7* homolog for *FLAVONOID-3*′*-MONOOXYGENASE (F3*′*H, PETAX027750_Contig1)*. The encoded enzymes are responsible for the hydroxylation of (colorless) dihydroflavonols in the 3′ and 5′ positions for the production of the tri- and di-substituted anthocyanin derivatives, respectively (Table 1; Bogs et al., 2006). Also the expression of *JAF13* (*PETAX002230_Contig1*), encoding for a bHLH transcription factor specifically involved in the control of anthocyanin synthesis (Quattrocchio et al., 1998), increased in *VvWRKY26* expressing plants, although with a low fold change (FC = 2.11; Supplementary File 1). Other genes involved in secondary metabolic processes resulted modulated (Table 1). Among these, noteworthy are the *4-COUMARATE-CoA LIGASE* (*4CL, PETAX000309_Contig241*) and *p-COUMARATE 3-HYDROXYLASE (C3H, PETAX000309_Contig83*) of the phenylpropanoid pathway.

Ultimately, even cell wall metabolism resulted as being affected by *VvWRKY26*, as revealed by the modulation of several pectinesterases, an expansin (*PETAX032024_Contig1*) and an endoβ-1,4-glucanase (*PETAX019178_Contig1*) (Supplementary File 1).

**Figure 4:**
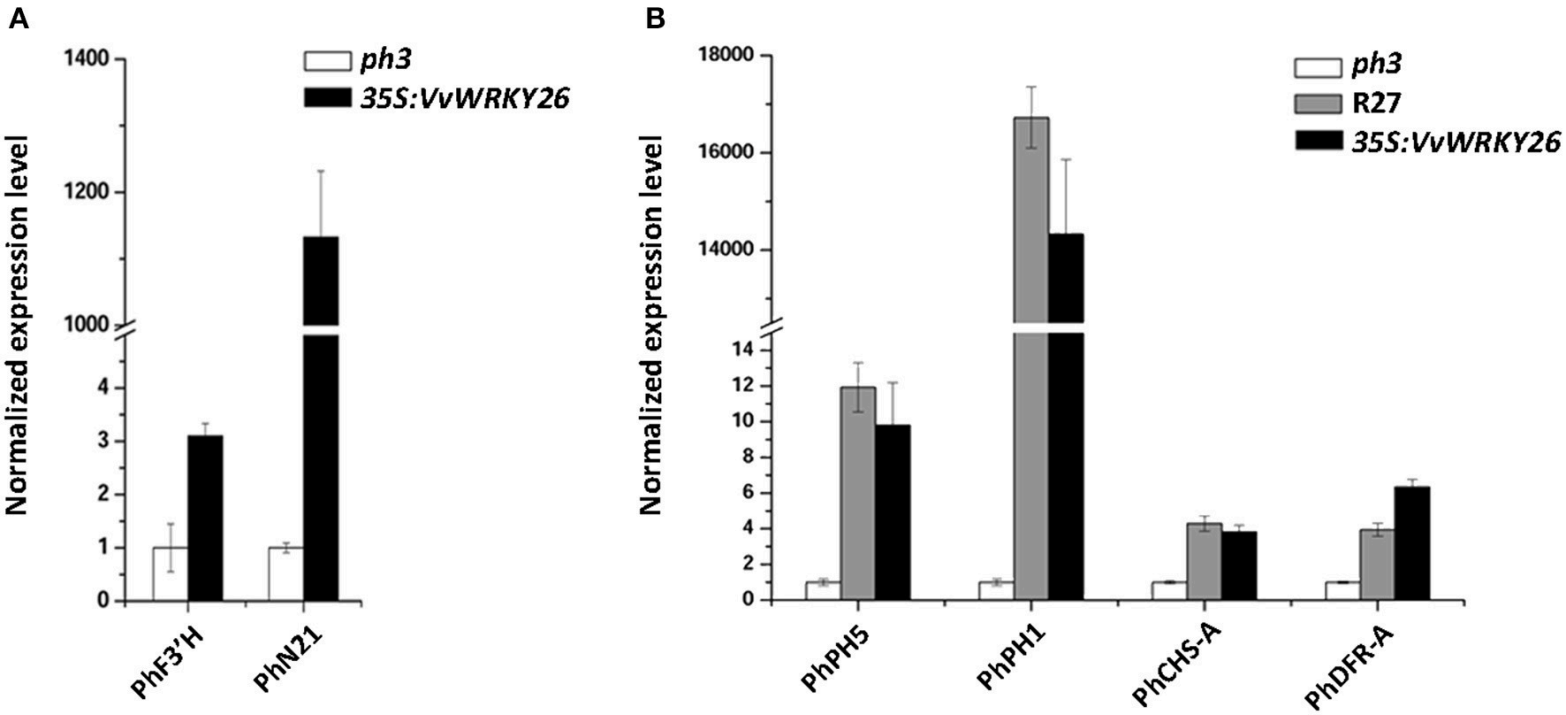
**Expression analyses in petunia petals by qPCR. (A)** Expression analysis of *F3*′*H* and *N21* in the *ph3* mutant and in *VvWRKY26* expressing lines as confirmation of the microarray results. **(B)** Expression analysis of structural genes related to vacuolar acidification (PhPH5 and PhPH1) and to anthocyanin synthesis (PhCHS-A and PhDFR-A) in the untransformed R27 and *ph3* lines and *VvWRKY26* expressing plants. In all analyses the data correspond to the mean ± SE of three biological replicates (corresponding to lines 1, 2, and 5; Supplementary Figure 2) relative to an *ACTIN* housekeeping control and normalized against the *ph3* mutant value. Abbreviations correspond to: *PhF3*′*H, FLAVONOID-3*′*-MONOOXYGENASE; PhN21, NODULIN MTN21-LIKE PROTEIN; PhPH5, H*^+^ *P*_3*A*_*-ATPASE; PhPH1, P*_3*B*_*-ATPASE; PhCHS-A, CHALCONE SYNTHASE A; PhDFR-A, DIHYDROFLAVONOL 4-REDUCTASE A*.

Our microarray analysis was validated comparing by qPCR the expression level of two modulated genes, *N21* and *F3*′*H* in *VvWRKY26* complemented lines and in the *ph3* mutated lines (Figure 4A).

These transcriptomic findings indicate that the heterologous expression of *VvWKY26* impacted multiple metabolic processes in petunia and, above all, those related to transport and flavonoid synthesis. However, it did not provide an exhaustible explanation for the phenotypic changes observed in petals specifically regarding vacuolar acidification and anthocyanin biosynthesis. We therefore analyzed by qPCR the expression level of the pH structural genes *PH5* and *PH1* and of the anthocyanin-related genes *CHS-A* and *DFR-A* in the transgenic line with highest levels of *VvWRKY26* expression (line 2), in the wild type R27 and in the *ph3* mutant lines (Figure 4B). The analysis revealed that the ectopic expression of *VvWRKY26* in *ph3* background induced the expression of *PH5, PH1, DFR-A* and *CHS-A* in petunia petals at comparable levels with the wild type R27 restoring the *ph3* mutation. qPCR data highlighted a difference, not detected by microarray analysis, probably due to its lower sensitivity, in the *DFR-A* and *CHS-A* expression level between mutant and complemented petunia lines. Otherwise, the expression of the pH-related genes *PH5* and *PH1* was not detected by microarray because they are not represented among chip probes.

These results support multiple roles for VvWRKY26 in petunia indicating that, above all, it is likely involved in the regulatory network underlying both pigmentation and vacuolar acidification.

### *VvWRKY26* expression analysis in grapevine

**Figure 5:**
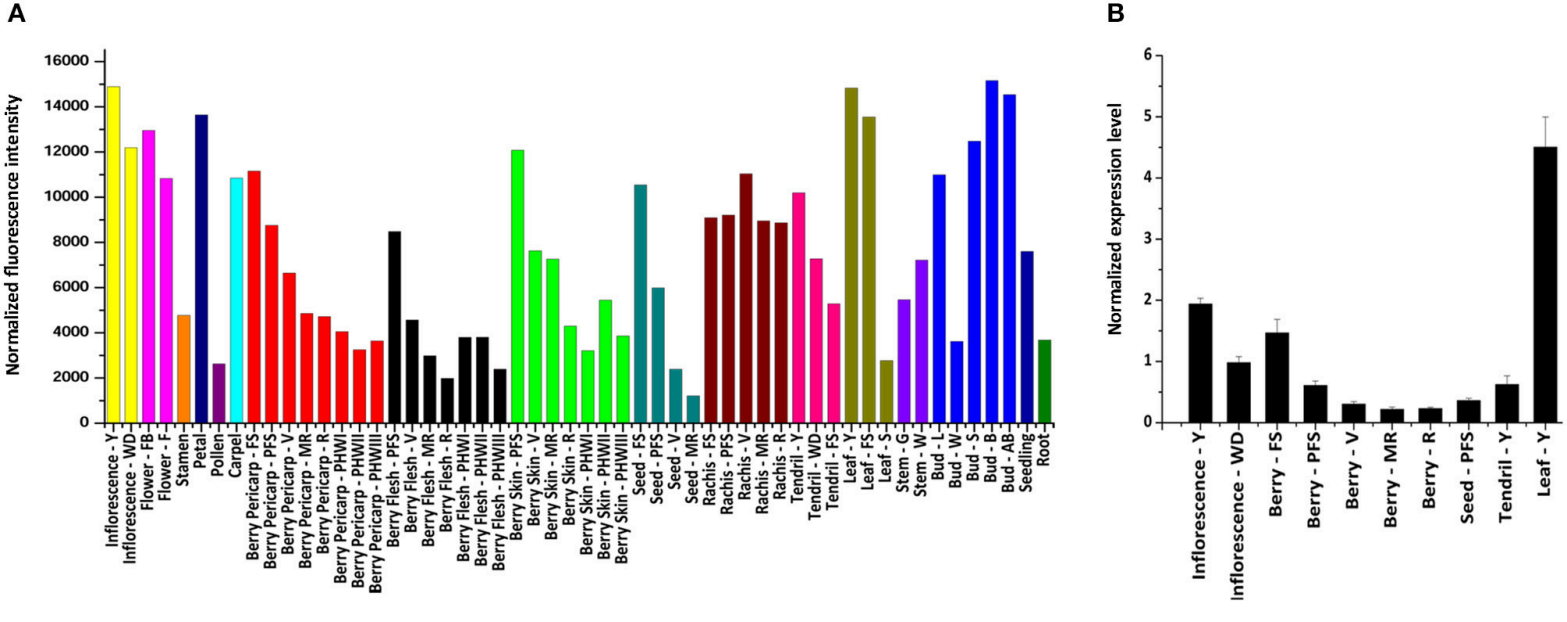
*****VvWRKY26*** expression profile in grapevine. (A)** *VvWRKY26* expression profile in the *V. vinifera* cv. Corvina atlas in 45 organs/tissues during development. **(B)** Expression analysis of *VvWRKY26* by qPCR in selected organs/stages of development of cv. Corvina Data, relative to *VvUBIQUITIN1* control, are the mean of three biological replicates ± SE. For both analyses the abbreviations after organ correspond to: FS, fruit set; PFS, post fruit set; V, véraison; MR, mid-ripening; R, ripening; Bud - L, latent bud; Bud - W, winter bud; Bud - S, bud swell; Bud - B, bud burst; Bud - AB, bud after burst; Inflorescence - Y, young; Inflorescence - WD, well-developed; Flower - FB, flowering begins; Flower - F, flowering; Tendril - Y, young; Tendril - WD, well-developed; Tendril - FS, mature; Leaf - Y, young; Leaf - FS, mature; Leaf - S, senescing leaf; Stem - G, green; Stem - W, woody.

The expression of *VvWRKY26* was determined by consulting the global gene expression map of *Vitis vinifera* cv. Corvina (Figure 5A; Fasoli et al., 2012). *VvWRKY26* is expressed in many grapevine organs with the highest levels in inflorescence, flower, petal and carpel, in young leaf, in rachis and tendril and in all stages of bud development, except for winter–bud. In berry tissues (whole pericarp, flesh and skin) and seed, *VvWRKY26* transcripts were mainly detected at the earliest stages of development (fruit set and post fruit set) and declined thereafter. The *VvWRKY26* expression pattern was confirmed by qPCR in a reduced set of Corvina organs, as berry pericarp throughout development (fruit set, post fruit set, véraison, mid-ripening, ripening), seed (post fruit set), young leaf, tendril and in young and well-developed inflorescence (Figure 5B).

**Figure 6:**
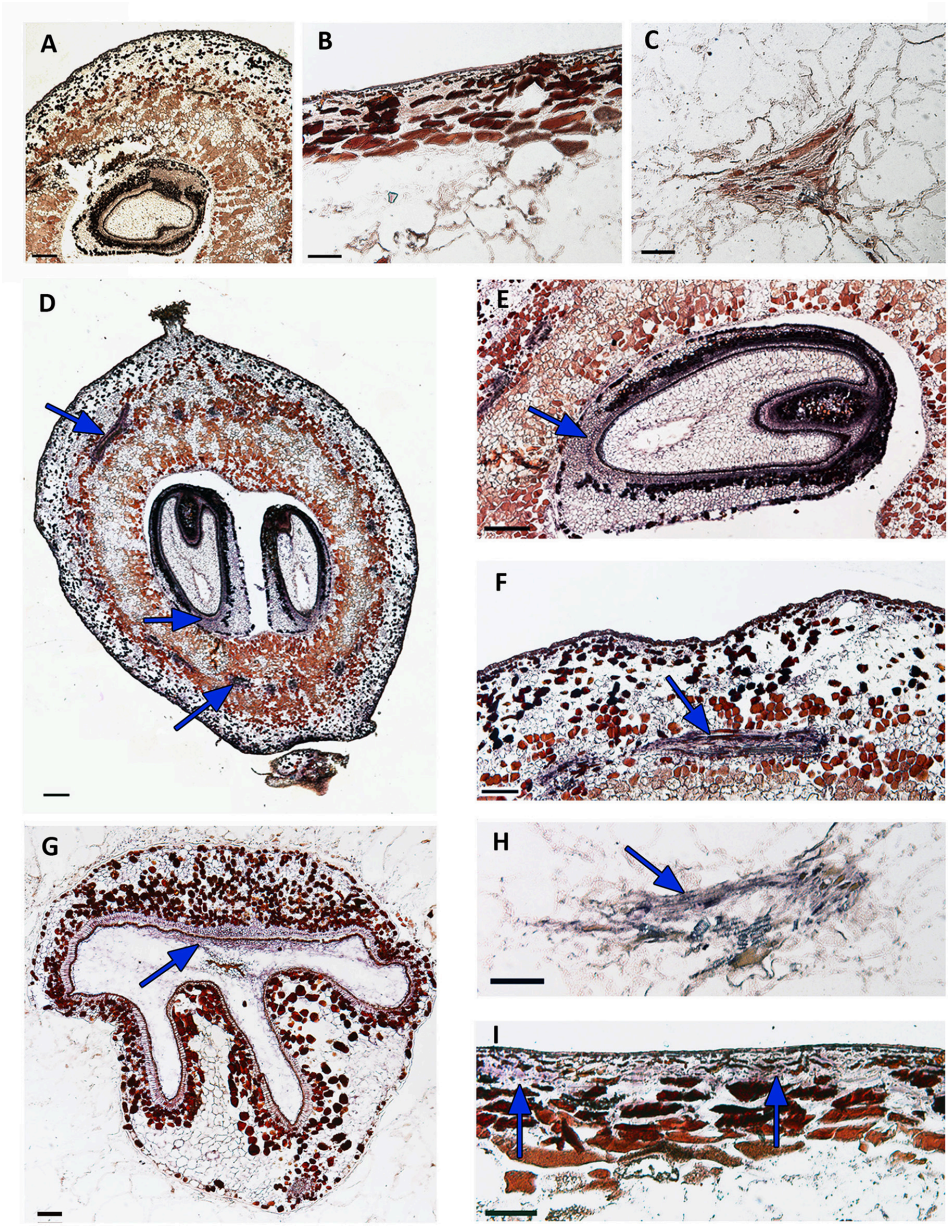
**Localization of ***VvWRKY26*** transcripts by ***in situ*** hybridization in berry at fruit set and post véraison stages**. Sections of berry hybridized with a *VvWRKY26* RNA sense probe as negative control did not show any significant signals **(A–C)**. The antisense probe detected *VvWRKY26* transcripts, resulting in a violet-gray coloration **(D–I). (A–C)** represent the hybridization of longitudinal section of berry at post véraison, a magnification of skin and of a vascular bundle, respectively, with a *VvWRKY26* RNA sense probe (negative control). **(D–F)**, representing the hybridization of the berry at fruit set, report the signals in the longitudinal section of the whole berry **(D)**, in the inner integument of the seed coat **(E)** and in cells surrounding the vascular bundles **(F)**. At this developmental stage no signal was detected in the skin. **(G–I)**, representing the hybridization of the berry at post véraison, report the signals in the inner integument of a seed coat (transversal section, **G**), in the phloematic cells **(H)** and in the epidermal cell layer of the skin **(I)**.

In an attempt to gain insights into its role during berry development, we localized in detail the expression sites of *VvWRKY26* in berry tissues of Corvina at fruit set and post véraison by performing *in situ* hybridization (Figure 6). Hybridization with the *VvWRKY26* sense probe as control showed no background staining or signal (Figures 6A–C). The analysis revealed that the expression of *VvWRKY26* in seed is mainly localized in the cellular layer constituting the inner integument in both early (Figures 6D,E) and late (Figure 6G) developmental stages. In berry *VvWRKY26* expression was mainly localized in cells surrounding the vascular bundles of the mesocarp at fruit set (Figures 6F,G). A similar and even clearer signal was also detected later in development (post véraison), revealing mRNA localization specifically in the phloematic cells of vasculature (Figure 6H). Moreover, only at post véraison a hybridization signal was also detected in the epidermal cell layer of the exocarp (Figure 6I).

Our results indicated that *VvWRKY26* is widely expressed in grapevine organs mainly during early developmental stages. In berry its expression is localized in the vascular bundles of the mesocarp, while in seed in the inner integument of the seed coat.

### Correlation analysis of gene expression by the grapevine database VTCdb

In order to discover genes co-expressed with *VvWRKY26* in berry and possibly representing its targets or collaborators, we investigated the grapevine co-expression database VTCdb, encompassing over 800 public microarray datasets related to *Vitis vinifera* and representing the transcriptome changes in a wide range of biological and environmental conditions (Wong et al., 2013). The use of such a platform allowed to visualize the expression profile of genes highly correlated with *VvWRKY26* transcriptional pattern over a wide range of experimental and/or environmental conditions.

Considering a PCC > 0.7, we generated a list of 114 co-expressed genes (Supplementary File 2), that were automatically annotated using V1 version of the 12X grapevine genome and manually classified in functional categories based on their known or putative function (Supplementary Figure 4). The most highly represented category was constituted by transport-related genes (20.2%) (Table 2). Interestingly, these included the homologs of *PhPH5/AtTT13* (*VIT_09S0002G00130*) and *PhPH1* (*VIT_07S0191G00260*) involved in vacuolar acidification (Verweij et al., 2008; Faraco et al., 2014; Appelhagen et al., 2015). We also identified three genes related to K^+^ transport as the homolog of the Arabidopsis *NHX1*, an anion exchanger involved in vacuolar K^+^ fluxes mediating stomatal aperture (*VIT_14S0128G00020*; Andrés et al., 2014), the K^+^ efflux antiporter 3 (*KEA3, VIT_00S0282G00020*) belonging to the cation/proton antiporters-2 superfamily in Arabidopsis (Kunz et al., 2014) and the K^+^ uptake permease 6 (*KUP6, VIT_14S0066G01580*) (Kim et al., 1998). In the same category there are two members of the nucleobase-ascorbate transporter (NAT) family, *NAT6* (*VIT_17S0000G07850*) and *NAT11* (*VIT_00S0813G00010*), putatively involved in long-distance transport due to their preferential expression in vascular tissues of many Arabidopsis organs (Maurino et al., 2006) and the homologs of *PHT2-1* (*VIT_00S0291G00060*) and *PHT1-9* (*VIT_18S0122G00780*), representing two Arabidopsis inorganic phosphate transporters (Versaw and Harrison, 2002; Lapis-Gaza et al., 2014). Among the other co-expressed genes, we identified two MATE transporters (*VIT_08S0056G01120*; *VIT_08S0056G01000*), an ABC transporter (*VIT_13S0074G00690*) and an anthocyanin membrane protein 1 (*ANM1, VIT_08S0007G03570*), whose detailed functions are still unknown.

**Table 2:** **List of transport-related genes resulting as highly correlated with ***VvWRKY26*** (PCC > 0.7) in the correlation analysis of global gene expression performed using the grapevine transcriptomic platform VTCdb**.

**ID_Code**	**Abbreviation**	**Description**	**PCC^a^**
VIT_09s0002g00130	VvPH5 (VvAHA10)	H+-ATPase AHA10	0.79
VIT_14s0128g00020	NHX1	Na+/H+ exchanger	0.77
VIT_00s0291g00060	PHT2-1	Inorganic phosphate transporter 2-1	0.77
VIT_03s0038g00430	EDS5	Enhanced disease susceptibility 5	0.76
VIT_08s0056g01000	MATE	MATE efflux family protein	0.76
VIT_00s2279g00010	LEM3	ligand-effect modulator 3	0.76
VIT_18s0122g00780	PHT1-9	Inorganic phosphate transporter 1-9	0.75
VIT_14s0066g01580	KUP6	K+ uptake permease 6	0.75
VIT_17s0000g07850	NAT6	Nucleobase-ascorbate transporter 6	0.74
VIT_03s0063g02020	TIC62	Tic Complex Tic62 Subunit	0.74
VIT_00s1667g00010	LEM3	ligand-effect modulator 3	0.74
VIT_13s0074g00690	ABCG22	ABC transporter G member 22	0.74
VIT_00s0304g00020	UNE2	Unfertilized embryosac 2	0.74
VIT_16s0098g01290	Major Facilitator Superfamily protein	Major Facilitator Superfamily protein	0.73
VIT_00s0282g00020	KEA3	K+ efflux antiporter 3	0.72
VIT_01s0011g00600	APE2	Acclimation of photosynthesis to environment	0.72
VIT_00s0477g00070	VvPH1	Mg2+-importing ATPase	0.72
VIT_08s0007g03570	ANM1	Anthocyanin membrane protein 1	0.72
VIT_04s0008g06850	BT1	Biopterin transport-related protein 1	0.71
VIT_12s0059g02260	GLR5	Glutamate receptor 5	0.71
VIT_03s0097g00510	OPT4	Oligopeptide transporter 4	0.71
VIT_08s0056g01120	MATE	MATE efflux family protein	0.71
VIT_00s0813g00010	NAT11	Nucleobase-ascorbate transporter 11	0.71

a *PCC, Pearson's correlation coefficient*.

Among the genes not grouped in this category but highly co-expressed with *VvWRKY26* we found the R2R3-MYB repressor *MYBC2-L1* (*VIT_01S0011G04760*), recently demonstrated to influence pigmentation and cell vacuolar pH when expressed in petunia petals, and anthocyanin and PA biosynthesis in grapevine (Cavallini et al., 2014, 2015; Huang et al., 2014). We also found *MYC1* (*VIT_07S0104G00090*), a bHLH factor involved in the regulation of flavonoid synthesis (Hichri et al., 2010; Matus et al., 2010).

Another gene worth mentioning for the highest correlation of expression with *VvWRKY26* is *UV RESISTANCE LOCUS 8* (*UVR8*), a photoreceptor that induces changes in gene expression after absorption of UV-B rays mediating the photomorphogenic response (Binkert et al., 2014; Carbonell-Bejerano et al., 2014; Liu et al., 2015; Yin et al., 2015; Loyola et al., 2016).

Other two highly represented groups are related to cellular homeostasis and transcription factor activity, constituting 10.5% and 8.8% of the 114 genes, respectively. Many other functional categories were identified as almost equally represented. However, apart from the transport-related genes, it was difficult to find a putative functional association between all the other co-expressed genes.

The high correlation of expression of genes related to transport and vacuolar metabolism provides a first insight into the possible role exerted by VvWRKY26 in grapevine and indicates putative functional similarity with the petunia ortholog PH3. Moreover, the identification of many functional categories suggests that VvWRKY26 might also function in other physiological or stress-related pathways and therefore perform multiple functions.

### Transcriptomic analysis of *Vitis vinifera* cv. sultana leaves transiently over-expressing *VvWRKY26*

To identify potential target genes of VvWRKY26 in the native species we transfected six grapevine cv. Sultana plantlets with a *35S:VvWRKY26* construct by vacuum *Agrobacterium*-mediated infection. As control, six plantlets were transformed with a construct containing a non-coding sequence. Six days after infection the over-expression of *VvWRKY26* that occurred was verified by RT-PCR and qPCR analysis (Supplementary Figures 5A–B). Using an Agilent platform we compared the leaf transcriptomes of three selected over-expressing and control lines showing comparable high and low *VvWRKY26* expression level, respectively (Supplementary Figure 5B; Microarray Gene Expression Omnibus database accession GSE86891). Performing a *t*-test analysis with a Pearson's correlation value of 0.05, we identified 983 differentially expressed genes that were annotated using the V1 version of the 12X draft release of the grapevine genome and by manual BLAST analysis (Supplementary File 3). As putative targets of VvWRKY26 we focused on up-regulated genes with a fold change ≥ 2 that we distributed into 18 Gene Ontology functional categories (Supplementary File 3; Supplementary Figure 6). Those genes with no similarity to known sequences or function (no hit or unknown protein) were removed from the subset.

The transient over-expression of *VvWRKY26* affects a wide range of cellular processes (Supplementary File 3). Secondary metabolism and Transport categories were nonetheless well represented (Table 3). As observed in petunia, among genes related to transport, we found two genes encoding for ammonium transporters (*VIT_07S0031G02950* and *VIT_07S0031G02990*), and two nodulins (*VIT_17S0000G00830* and *VIT_04S0044G00380*). In addition, different ion transporters, a MATE efflux protein (*VIT_08S0007G08200*) and an ABC transporter (*VIT_01S0011G04670*) were modulated in their expression level, according to the proposed role of VvWRKY26 in transport. Moreover, carboxyesterases, lipoxygenases and patatins, putatively involved in lipid metabolism resulted as slightly up-regulated by the transgene supporting a role of VvWRKY26 in membrane remodeling and in maintaining vacuolar homeostasis.

**Table 3:** **Sub-set of up-regulated genes in ***VvWRKY26*** over-expressing Sultana plantlets involved in Secondary metabolism, Transport, and Lipid metabolism**.

**ID_Code**	**Description**	**Functional Category**	**FC^a^**	
VIT_11s0052g00790	Serine carboxypeptidase SCPL9	Secondary Metabolic Process	18.93	
VIT_15s0046g00170	VvMYBPA1	Secondary Metabolic Process	6.64	
VIT_13s0106g00550	Flavonol 3-sulfotransferase	Secondary Metabolic Process	5.76	
VIT_03s0091g00040	Hydroxybenzoate/hydroxycinnamate UDP-glucosyltransferase (VvGT1)	Secondary Metabolic Process	5.15	
VIT_19s0014g01970	Flavonol 3-O-glucosyltransferase	Secondary Metabolic Process	4.58	
VIT_14s0068g00930	Chalcone synthase 1 (VvCHS1)	Secondary Metabolic Process	4.47	
VIT_06s0009g02920	Flavonoid 3′,5′-hydroxylase (VvF3′5′H)	Secondary Metabolic Process	4.43	^*^
VIT_16s0050g01680	UDP-glucose: anthocyanidin 5,3-O-glucosyltransferase	Secondary Metabolic Process	3.71	
VIT_18s0001g11430	Flavonoid 3′-monooxygenase	Secondary Metabolic Process	3.56	^*^
VIT_18s0072g00920	Caffeate 3-O-methyltransferase 1 (VvCOMT1)	Secondary Metabolic Process	3.24	
VIT_02s0087g00490	10-deacetylbaccatin III 10-O-acetyltransferase	Secondary Metabolic Process	3.04	
VIT_00s0361g00020	Anthocyanidin reductase (VvANR)	Secondary Metabolic Process	2.95	
VIT_16s0050g01590	UDP-glucose: anthocyanidin 5,3-O-glucosyltransferase	Secondary Metabolic Process	2.87	
VIT_16s0050g01580	UDP-glucose: anthocyanidin 5,3-O-glucosyltransferase	Secondary Metabolic Process	2.75	
VIT_06s0004g02620	Phenylalanine ammonia-lyase	Secondary Metabolic Process	2.67	
VIT_18s0001g14310	Flavanone-3-hydroxylase 2 (VvF3H2)	Secondary Metabolic Process	2.60	
VIT_10s0003g03750	9-cis-epoxycarotenoid dioxygenase 2	Secondary Metabolic Process	2.48	
VIT_08s0007g05360	Strictosidine synthase	Secondary Metabolic Process	2.22	
VIT_13s0064g00390	Polyamine oxidase	Secondary Metabolic Process	2.21	
VIT_01s0011g02960	Leucoanthocyanidin reductase 1 (VvLAR1)	Secondary Metabolic Process	2.13	
VIT_02s0025g00700	Aluminum-activated malate transporter 9	Transport	7.49	
VIT_05s0020g03970	Sulfate transporter 12	Transport	6.09	
VIT_17s0000g00830	Nodulin MtN3	Transport	4.19	^*^
VIT_07s0031g02990	Ammonium transporter 2	Transport	3.08	^*^
VIT_14s0006g02550	Non-specific lipid-transfer protein 2	Transport	3.02	
VIT_19s0027g01880	Amino acid transport protein	Transport	3.01	
VIT_07s0031g02950	Ammonium transporter 2	Transport	2.82	^*^
VIT_18s0001g11470	CYP82A3	Transport	2.82	
VIT_18s0001g11490	CYP82C1p	Transport	2.82	
VIT_08s0007g08200	MATE efflux family protein	Transport	2.81	
VIT_04s0044g00380	Nodulin MtN21	Transport	2.77	^*^
VIT_19s0027g01870	Amino acid permease	Transport	2.45	
VIT_03s0038g02930	Tetracycline transporter protein	Transport	2.32	
VIT_19s0177g00280	DNAJ heat shock N-terminal domain-containing	Transport	2.32	
VIT_15s0048g01210	Subtilisin serine endopeptidase	Transport	2.28	
VIT_01s0011g04670	ABC Transporter 7	Transport	2.21	
VIT_00s0827g00020	Dicarboxylate/tricarboxylate carrier	Transport	2.19	
VIT_17s0000g08530	Boron transporter-like protein 1	Transport	2.11	
VIT_03s0091g01290	Serine carboxypeptidase S10	Lipid Metabolic Process	6.86	
VIT_03s0063g00800	Carboxyesterase 12	Lipid Metabolic Process	2.84	
VIT_06s0080g00310	Esterase/lipase/thioesterase family protein	Lipid Metabolic Process	2.61	
VIT_06s0004g01510	Lipoxygenase	Lipid Metabolic Process	2.60	^*^
VIT_16s0022g02430	Carboxyesterase 20	Lipid Metabolic Process	2.54	
VIT_06s0004g01460	Lipoxygenase 2	Lipid Metabolic Process	2.34	^*^
VIT_15s0048g02720	Fatty acid elongase	Lipid Metabolic Process	2.32	
VIT_18s0001g10900	Patatin	Lipid Metabolic Process	2.24	
VIT_16s0022g02440	Carboxyesterase 20	Lipid Metabolic Process	2.23	
VIT_18s0001g10910	Patatin	Lipid Metabolic Process	2.18	
VIT_16s0100g00010	Carboxyesterase 20	Lipid Metabolic Process	2.09	

* *Defines those genes whose function can be associated with that of specific genes up-regulated in petunia flowers due to the ectopic expression of VvWRKY26*.

a *FC, fold change*.

The Secondary metabolism category was widely represented by genes encoding enzymes acting in flavonoid biosynthesis (Table 3). The transcription factor *MYBPA1* (*VIT_15S0046G00170*), the regulator of PA biosynthesis, resulted as being one of the most induced genes by *35S:VvWRKY26* (Bogs et al., 2007). Consistently, we observed the modulation of several structural flavonoid genes like *CHS1* (*VIT_14S0068G00930*), *F3H2* (*VIT_18S0001G14310*), *F3*′*5*′*H* (*VIT_06S0009G02920*) and *F3*′*H* (*VIT_18S0001G11430*). Moreover, the transcriptional analysis revealed the up-regulation of many genes specifically involved in proanthocyanidin synthesis, i.e., *ANTHOCYANIDIN REDUCTASE* (*ANR, VIT_00S0361G00020*), *LEUCOANTHOCYANIDIN REDUCTASE 1* (*LAR1, VIT_01S0011G02960*), and the recently characterized *HYDROXYBENZOATE/HYDROXYCINNAMATE UDP-GLUCOSYLTRANSFERASE* (*GT1, VIT_03S0091G00040*) (Bogs et al., 2005; Khater et al., 2012). The laccase encoding transcript *VIT_18s0122g00250*, putatively involved in the oxidative polymerization of flavan-3-ols and strongly induced in *35S:VvWRKY26* plantlets, may also be included in this proanthocyanidin-related gene list. Lastly, *SERINE CARBOXYPEPTIDASE SCPL9* (*VIT_11S0052G00790*) resulted as significantly induced with an FC of 18.92. This gene was also recently identified in the transcriptomic screening of grapevine hairy roots over-expressing *MYBPA1* and *MYBPA2* (Khater et al., 2012). Based on these results we suggest that in grapevine VvWRKY26 functions as a regulator of the flavonoid pathway specifically targeting the proanthocyanidin branch.

In our analysis we also found several genes belonging to the stress response category and different senescence-associated proteins, supporting a role of VvWRKY26 in plant response to abiotic stresses as previously proposed (Guo et al., 2014; Wang L. et al., 2014).

**Figure 7:**
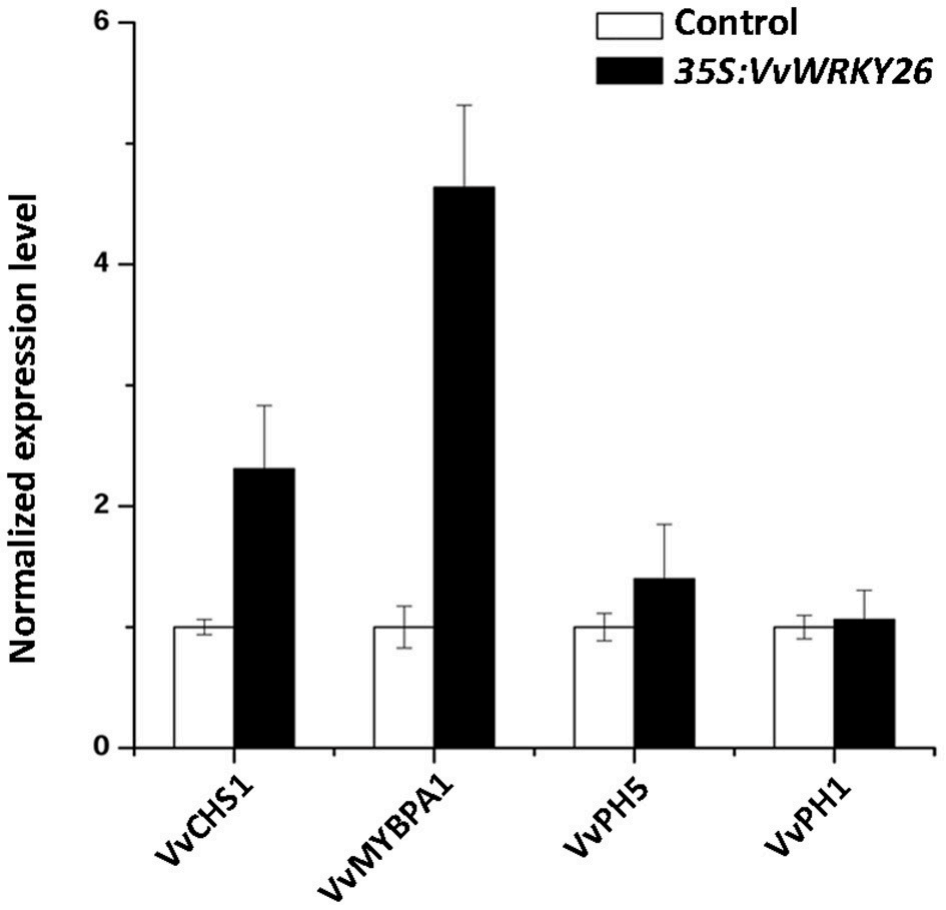
**Expression analysis of ***CHS1, MYBPA1, PH5***, and ***PH1*** by qPCR in the control lines and ***VvWRKY26*** expressing Sultana plantlets**. The data correspond to the mean ± SE of three biological replicates relative to the *VvUBIQUITIN1* control and normalized against each control value. Abbreviations correspond to: *VvCHS1, CHALCONE SYNTHASE 1; VvMYBPA1, MYB regulator of PA biosynthesis; VvPH5, H*^+^ *P*_3*A*_*-ATPASE; VvPH1, P*_3*B*_*-ATPASE*.

Our microarray analysis was validated by qPCR comparing the expression level of *MYBPA1, CHS1, PH5*, and *PH1*, in control and *VvWRKY26* transiently over-expressing lines (Figure 7).

Taken together, the results obtained by *VvWRKY26* over-expression in grapevine partially confirmed the functional analysis conducted in petunia, suggesting its involvement in transport, vacuole-associated metabolisms and flavonoid biosynthesis, specifically in control of the PA pathway.

## Discussion

### VvWRKY26 is the grapevine ortholog of PhPH3, AtTTG2, and BnTTG2

The WRKY proteins represent one of the largest families of transcription factors in the plant kingdom. Since the first identification in sweet potato (Ishiguro and Nakamura, 1994), the regulative functions of members of this family have mainly been associated with plant response to biotic and abiotic stresses. However, a remarkable number of WRKY proteins have also important roles in development and in the control of secondary metabolic pathways (Schluttenhofer and Yuan, 2015). This is the case of the well-characterized TTG2 of Arabidopsis that is a regulator of seed and trichome development (Johnson et al., 2002; Garcia et al., 2005; Ishida et al., 2007; Gonzalez et al., 2016). Two WRKY factors extremely close to AtTTG2 have recently been characterized, PH3 of *P. hybrida*, a novel regulator of vacuolar acidification in petals (Verweij et al., 2016), and TTG2 of *Brassica napus* involved in response to salt stress and in trichome development (Li et al., 2015).

In this study, we isolated and characterized *VvWRKY26* as the closest grapevine homolog to Arabidopsis TTG2 and petunia PH3. Our phylogenetic analysis showed that *VvWRKY26* together with AtTTG2, PhPH3 and BnTTG2 form a distinct clade of WRKYs belonging to group Ia, defined by the presence of two WRKY domains and the C2H2-type zinc-finger motifs (Wang M. et al., 2014). Moreover, our phylogenetic tree based on the sequence similarity of the C-terminal WRKY domains also reflects the classification in subgroups established for the number of the WRKY domain in the protein and the type of zinc-finger motif (Eulgem et al., 2000; Zhang and Wang, 2005; Rushton et al., 2010), indicating a high degree of amino acidic conservation of the C-terminal WRKY domain within each subgroup. The importance of the C-terminal WRKY domain relies on its exclusive strong ability to recognize and bind the cognate W-boxes in the promoter of the target genes, in contrast to the N-terminal domain, that was shown to bind the DNA with low affinity (Brand et al., 2013). However, the N-terminal region is characterized by unique domains shared by members of the TTG2-like clade, suggesting that it may contribute to give specificity in the regulatory function of these WRKY factors.

VvWRKY26 showed high homology with PhPH3, sharing very similar β2, β3, and β4 strands necessary for the interaction with DNA as proposed by Brand et al. (2013). Moreover, both coding sequences harbor an intron between the WRKYGQK motif and the zinc finger that is typical of the C-terminal WRKY domain of group I WRKY proteins (Eulgem et al., 2000; Brand et al., 2013). This feature distinguishes *AtTTG2* and *BnTTG2* from the grapevine and petunia homologs. Interestingly, BnTTG2 can fulfill AtTTG2 regulative functions in trichome development when expressed in Arabidopsis (Li et al., 2015) and, on its part, AtTTG2 mimics PhPH3 in the control of vacuolar acidification when expressed in petunia (Verweij et al., 2016).

Moreover, both AtTTG2 and PhPH3 are part of a similar regulative and transcriptional network, in which the respective MBW complexes activate their expression and also require their interaction to activate the downstream pathways (Gonzalez et al., 2016; Verweij et al., 2016). In this study we provide evidence that VvWRKY26 is able to fulfill the functions of PhPH3 when expressed in petunia *ph3* mutant, and functional analogies were also found between VvWRKY26 and the homolog AtTTG2 regarding proanthocyanidin biosynthesis as indicated by the induction of numerous PA-related genes in Sultana plants over-expressing *VvWRKY26*. These observations suggest that all members of the TTG2-like clade are functionally orthologs and are likely part of a similar regulation mechanism, although they control pathways that are partially distinct in their endogenous species. The functional orthology might also depend on the promoter sequence of the target genes and therefore is achieved by both remarkable conservation within WRKY proteins between species and the cognate binding site (C/T)TGAC(T/C) of the W-boxes (Pesch et al., 2014). For Group I WRKY factors, it has been proposed that two different and consecutive W-box motifs in the promoter sequence might be necessary for the binding of both WRKY domains (Brand et al., 2013). Future identification of the target genes in grapevine and analysis of the DNA regulative regions will be helpful to elucidate the recognition mechanism of VvWRKY26 and to highlight analogies of the regulative mechanisms within the TTG2-like protein cluster.

### Evidence of an involvement of VvWRKY26 in vacuolar acidification and transport across the tonoplast

The sequencing of the grapevine genome and the consequent availability of about 30,000 predicted gene sequences (Jaillon et al., 2007) prompted the exploitation of regeneration and transformation tools to gain information about gene function in grapevine. However, due to the recalcitrant nature of many cultivars to the agrobacterium-mediated stable transformation and the long regeneration time of the transgenic plantlets, the stable transformation of grapevine is still time-consuming and difficult to perform. The ectopic expression of a grapevine gene in a heterologous species such as *P. hybrida*, representing a fast growing model, has previously proven to be a useful alternative for functional characterization (Bogs et al., 2006; Cavallini et al., 2014, 2015; Provenzano et al., 2014; Li et al., 2016). Moreover, many well-characterized mutants for genes involved in different biochemical and developmental pathways are available for this model species. In our case, the petunia line mutated in *PH3*, and thus showing a higher pH of corolla crude extracts and reduced anthocyanin content, was used to confirm the functional orthology between PhPH3 and VvWRKY26. In fact, ectopic expression of *VvWRKY26* caused an increase of *PH5* and *PH1* expression, the structural genes for vacuolar acidification, with the subsequent activation of pumping mechanisms leading to the decrease of pH in petal homogenates to wild type levels. Consistently, in our correlation analysis of gene expression using VTCdb, *VvWRKY26* gene expression was highly correlated with the grapevine homologs of the petunia *PH1* and *PH5*, whose functions as P-ATPase pumps were inferred by heterologous expression in petunia (Provenzano, 2011; Li et al., 2016).

However, the transient over-expression of *VvWRKY26* in grapevine cv. Sultana did not induce *PH1* and the expression of *PH5* resulted as only weakly increased, as shown by microarray and qPCR analyses. The high expression of the endogenous *VvWRKY26* in young leaves, as reported in the grapevine transcriptomic atlas (Fasoli et al., 2012) and by our qPCR, may have masked the effect of transgene over-expression. Alternatively, VvWRKY26 may require other co-factors for the activation of the downstream pathways, as recently demonstrated for the orthologs AtTTG2 and PhPH3 both cooperating with the respective WD protein of the MBW regulatory complex (Li et al., 2016; Verweij et al., 2016). It was previously shown that the grapevine MYB5a and MYB5b, participating in a MBW complex (Deluc et al., 2008; Hichri et al., 2010), are able to regulate cell pH when expressed in petunia petals (Cavallini et al., 2014). VvWRKY26 might require to be co-expressed with MYB5a and MYB5b to exert its biological function.

In the list of genes co-expressed with *VvWRKY26* from the VTCdb and in the lists of genes induced by the heterologous and ectopic expression of *VvWRKY26* in petunia and grapevine, respectively, the transport-related category was always highly represented indicating that VvWRKY26 might also impact the transport of anions/cations and other solutes across the tonoplast. In particular we identified many genes related to K^+^ transport and uptake. By exploiting VTCdb, the homolog of the Arabidopsis *NHX1*, a tonoplast-located K^+^/ H^+^exchanger required for K^+^ uptake into the vacuole of guard cells for stomatal opening was found (Andrés et al., 2014). In petunia the over-expression of *Ipomoea nil NHX1* counteracts the acidifying effect of PH1 and PH5 indicating that these NHX transporters influence the vacuolar pH in the opposite manner to the H^+^ ATPase pumps (Faraco et al., 2014). We also identified *KUP6*, a member of the K^+^ uptake permease family. In grapevine two KUP genes *VvKUP1* and *VvKUP2* have been characterized for their role in K^+^ accumulation likely associated with berry growth during development (Davies et al., 2006). Interestingly, in petunia *35S:VvWRKY26* induces two genes encoding for the Arabidopsis homolog *AtKAT2*, an inward rectifying K^+^ channel involved in stomatal opening (Sharma et al., 2013), and *AtHAK5*, a K^+^ transporter active in roots at very low external K^+^ concentration (Nieves-Cordones et al., 2014). Considering also the many additional transport-related genes, the information gained exploiting transcriptomic-based approaches suggested that VvWRKY26 participates in different transport regulatory mechanisms and controls the expression of many different genes generally contributing to maintaining vacuolar homeostasis. VvWRKY26 would thus act as regulator of the vacuolar pH and ion transport, controlling both H^+^ pumps that acidify the vacuolar lumen as well as additional targets that counterbalance the positive charge accumulation and dissipate the electrochemical gradient across the tonoplast.

### Evidence of an involvement of VvWRKY26 in flavonoid biosynthesis

The constitutive expression of *VvWRKY26* in petunia *ph3* line resulted in the induction of the flavonoid structural genes *CHS-A, F3*′*H, F3*′*5*′*H* and *DFR-A*, and in the restoration of the wild type anthocyanin content that was impaired in *ph3* mutant. This suggests that, in addition to the proposed role in vacuolar acidification and transport, VvWRKY26 might also be implicated in the control of the flavonoid biosynthetic pathway. The *HF2* gene encoding F3′5′H resulted as being the most up-regulated in the microarray analysis performed on *ph3/35S:VvWRKY26* complemented petunias. This enzyme is responsible for the hydroxylation of dihydroflavonols leading to the synthesis of the tri-substituted delfinidin-, malvidin- and petunidin-derivatives conferring a pink/purple pigmentation to the corolla. The genetic background of the *ph3* mutant plant is however characterized by mutations in *HF1* and *HF2* loci causing a non-functional F3′5′H proteins (Matsubara et al., 2006). Therefore, despite the *HF2* induction by VvWRKY26, petals of the fully complemented *35S:VvWRKY26* petunia plants have a reddish color typical of the cyanidin-based anthocyanin profile of the R27 background. The microarray analysis of petunia petals expressing *VvWRKY26* also revealed the weak induction of a bHLH regulator of the flavonoid pathway, *JAF13*, whose precise role in the pigmentation of petunia corollas still has to be fully elucidated (Quattrocchio et al., 1998; Montefiori et al., 2015).

A role of VvWRKY26 as a flavonoid regulator, which seems to narrow to the PA synthesis, can also be inferred from the analysis of the grapevine plantlets transiently over-expressing the transcription factor. The induction of *MYBPA1* (Bogs et al., 2007) and of its targets, i.e. the structural genes *ANR, LAR1*, and *GT1* (Bogs et al., 2005; Khater et al., 2012), indicates the ability of VvWRKY26 to influence the PA branch by controlling the expression of this specific PA regulator, although a direct control of the biosynthetic steps by VvWRKY26 cannot be ruled out. Our hypothesis is also reinforced by the up-regulation of *CHS1*, a chalcone synthase previously associated to PAs (Harris et al., 2013) and of several serine-carboxypeptidases, some of which, shown to be induced by MYBPA1 (Terrier et al., 2009). Finally, the up-regulation of both *F3*′*5*′*H* and *F3*′*H* genes in the leaves of the over-expressing grapevines, that were also found upregulated in *ph3/35S:VvWRKY26* petunias, confirms that VvWRKY26 regulates the hydroxylation steps of flavonoids in the endogenous species. The Arabidopsis homolog TTG2 was also shown to be a key regulator of PA accumulation, and *ttg2* mutants have a reduced deposition of PAs in the seed coat. AtTTG2 drives the PA vacuolar storage by directly regulating the ATPase proton pump *TT13* and the MATE vacuolar transporter *TT12* (Appelhagen et al., 2015; Gonzalez et al., 2016).

### Roles of VvWRKY26 in grapevine

The function of VvWRKY26 studied through the aforementioned approaches together with the analysis of its expression pattern in grapevine organs at various developmental stages allow us to speculate about the biochemical and developmental processes regulated in planta. We particularly focused on berry where *VvWRKY26* expression was high at early stages of fruit development and progressively declined at later stages. This general expression pattern is consistent with the hypothesized role in the regulation of berry acidification and PA biosynthesis, because the pH of berry homogenate is low at the first berry developmental stages and increases thereafter, and most of the PAs accumulate in berry tissues before the onset of ripening. *In situ* hybridization showed *VvWRKY26* expression in vascular bundles of berry pericarp at fruit set and during ripening, supporting its role in transport through development. The correlation with many transporters repeatedly encountered in our study suggests a specific involvement in the exchange of ions such as K^+^ between vessels and berry flesh. Grapes are very rich in potassium that is an essential macronutrient for growth and development. It is the main cation in must and wine and its concentration in the berry at harvest may affect pH and thus impact potential wine quality.

The high expression of *VvWRKY26* at the earliest stages of berry development also relies on its specific mRNA localization in the inner integument of the seed coat. This localization is consistent with the PA accumulation profile in the seed coat (Bogs et al., 2007), and the induction of many PA-related genes by *VvWRKY26* over-expression in Sultana plantlets corroborates its involvement in the deposition of PAs in seed tissues. The heterologous expression of *VvWRKY26* in petunia partially overcame the sterility in the *ph3* line, suggesting that both PhPH3 and VvWRKY26 might somehow be implicated in fertility determinacy. Such a role could be related to its regulative function on PA synthesis. In fact, it has been proposed that the Arabidopsis homolog TTG2 determines primarily cell elongation of the integument during seed development likely through the control of tannin synthesis that accumulates in these cell layers (Garcia et al., 2005). Dilkes et al. (2008) demonstrated that the role of AtTTG2 in seed endosperm development and PA synthesis has determinant implications in controlling the lethality of progenies derived by interploidy crosses. It is therefore plausible to hypothesize that VvWRKY26 functions as PA regulator and may also affect seed development and plant fertility in grapevine.

Clear signals of *VvWRKY26* mRNAs were specifically detected in the outer layers of berry skin after véraison when anthocyanins start to accumulate, indicating that VvWRKY26 may contribute to the synthesis of anthocyanins, a role that would mimic what has been observed in petunia corollas. The contribution of VvWRKY26 to the regulative mechanism of anthocyanin accumulation in grape and its role in relation to the known MBW complex of regulators (Walker et al., 2007; Hichri et al., 2010; Matus et al., 2010) controlling anthocyanin biosynthesis in grape berry skin deserve more attention.

Finally, considering that “beyond fruit” the expression of VvWRKY26 was detected in many plant organs, mainly during the early development, it must be postulated that its biochemical/developmental regulative roles are widespread in most organs of the grapevine. Future experiments are needed to address this issue.

### Final remarks

In this work we introduced VvWRKY26 as a new member of the TTG2-like protein clade. We shed light on VvWRKY26 multiple regulative functions in grapevine, suggesting above all that the high homology with AtTTG2 and PhPH3 can reflect similar regulative functions. Results obtained in the native species allowed us to propose VvWRKY26 as a putative regulator of vacuolar transport and acidification in grapevine but also to glimpse its involvement in other processes including flavonoid biosynthesis with a particular role in PA deposition. We demonstrated that VvWRKY26 could participate in the vacuolar acidification pathway through the regulation of an ATPase pump system likely conserved, at least partly, in grapevine, petunia and Arabidopsis. In grapevine seeds and berry skin, vacuolar acidification possibly represents a driving force for PA transport and accumulation in vacuoles, but in berry pulp this mechanism could play a key role in determining final berry juice acidity. Further investigations of these aspects would represent an important advance in the possibility to control the final berry quality traits for winemaking.

## Author contributions

AA and EC coordinated the study, discussed the results and wrote the manuscript. AA generated grapevine plantlets over-expressing *VvWRKY26*, performed both petunia and grapevine transcriptomic analyses, and performed berry fixation and embedding for *in situ* hybridization. EC performed the phylogenetic analysis, expression and correlation analyses, and characterized petunia *ph3/35S:VvWRKY26* complemented lines. SZ interpreted the microarray data, supervised the study and drafted the manuscript. LF generated petunia *ph3/35S:VvWRKY26* complemented lines. MB performed *in situ* hybridization. BR supervised *in situ* hybridization and contributed in drafting the manuscript. GT designed and supervised the study, interpreted the data and drafted the manuscript.

## Funding

This work benefited from the networking activities coordinated under the EU-funded COST ACTION FA1106 “An integrated systems approach to determine the developmental mechanisms controlling fleshy fruit quality in tomato and grapevine”, from FUR granted by the University of Verona to SZ and GBT and from “Ricerca scientifica Ex-60%” to BR.

### Conflict of interest statement

The authors declare that the research was conducted in the absence of any commercial or financial relationships that could be construed as a potential conflict of interest.
