# Supplementary figures and images for: A Grapevine TTG2-Like WRKY Transcription Factor Is Involved in Regulating Vacuolar Transport and Flavonoid Biosynthesis
Source: Front Plant Sci. 2017 Jan 5;7:1979. doi: 10.3389/fpls.2016.01979 (PMC5214514; doi:10.3389/fpls.2016.01979)

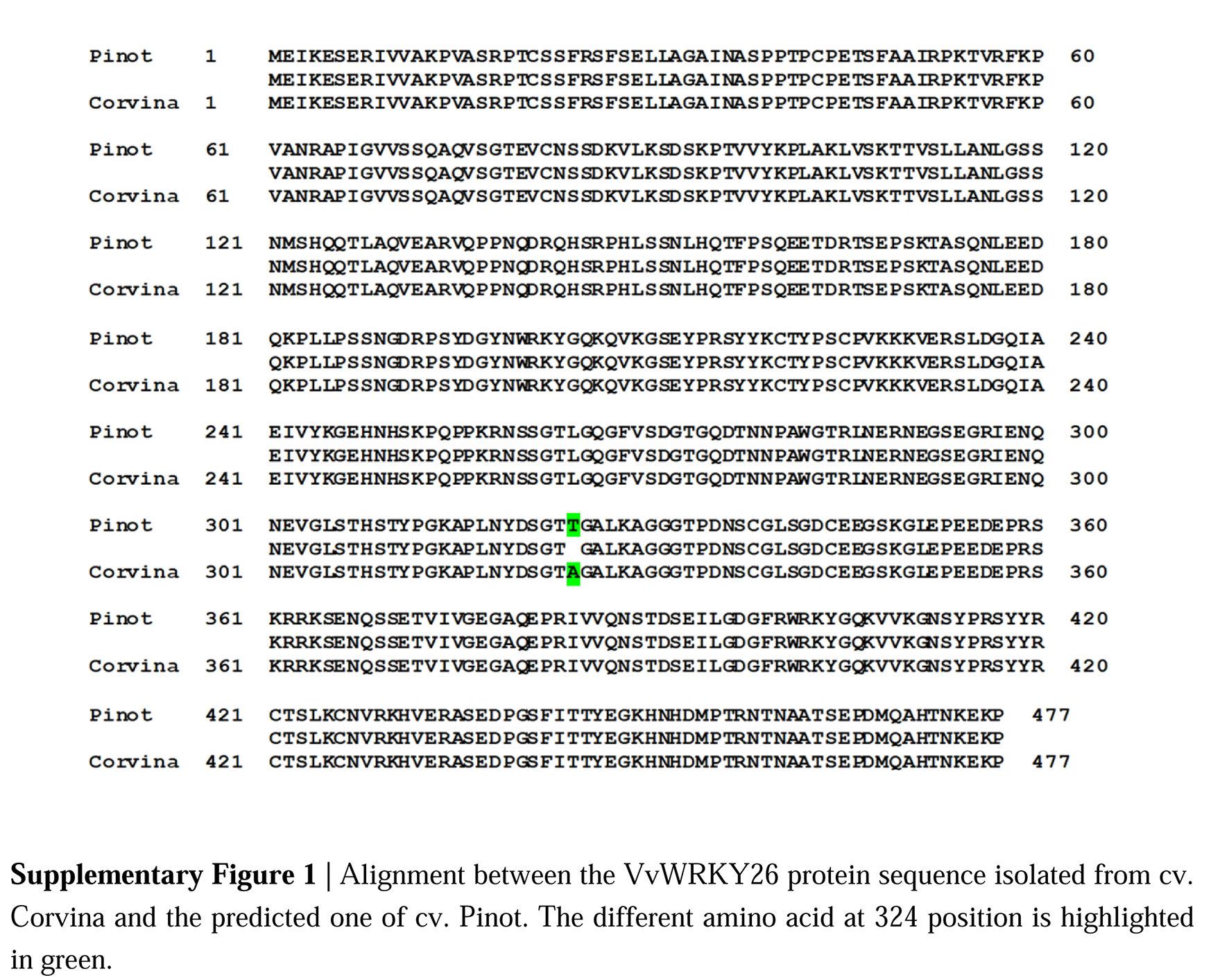

Supplement: Supplementary file 4 [file Image1.TIF]

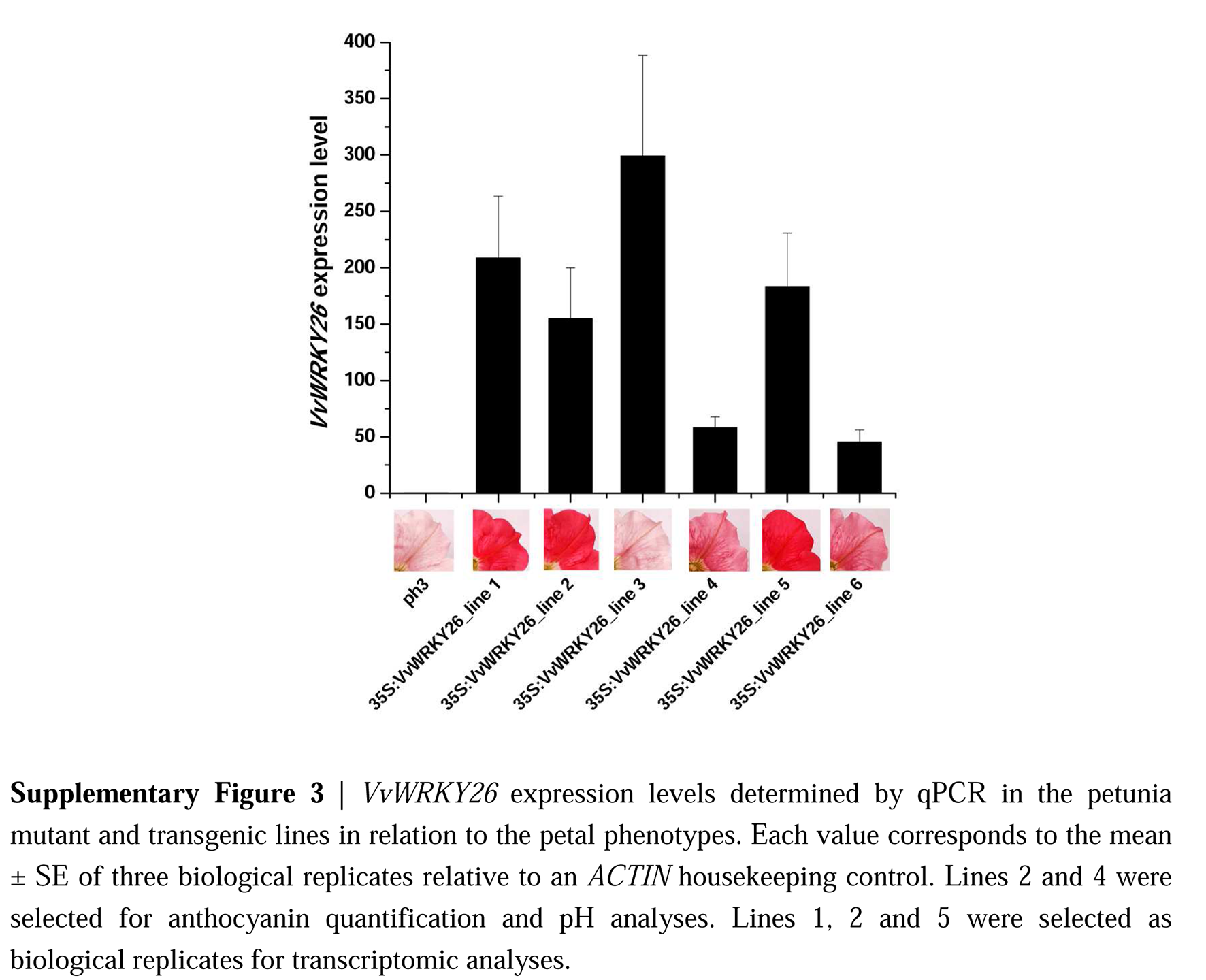

Supplement: Supplementary file 6 [file Image3.TIF]

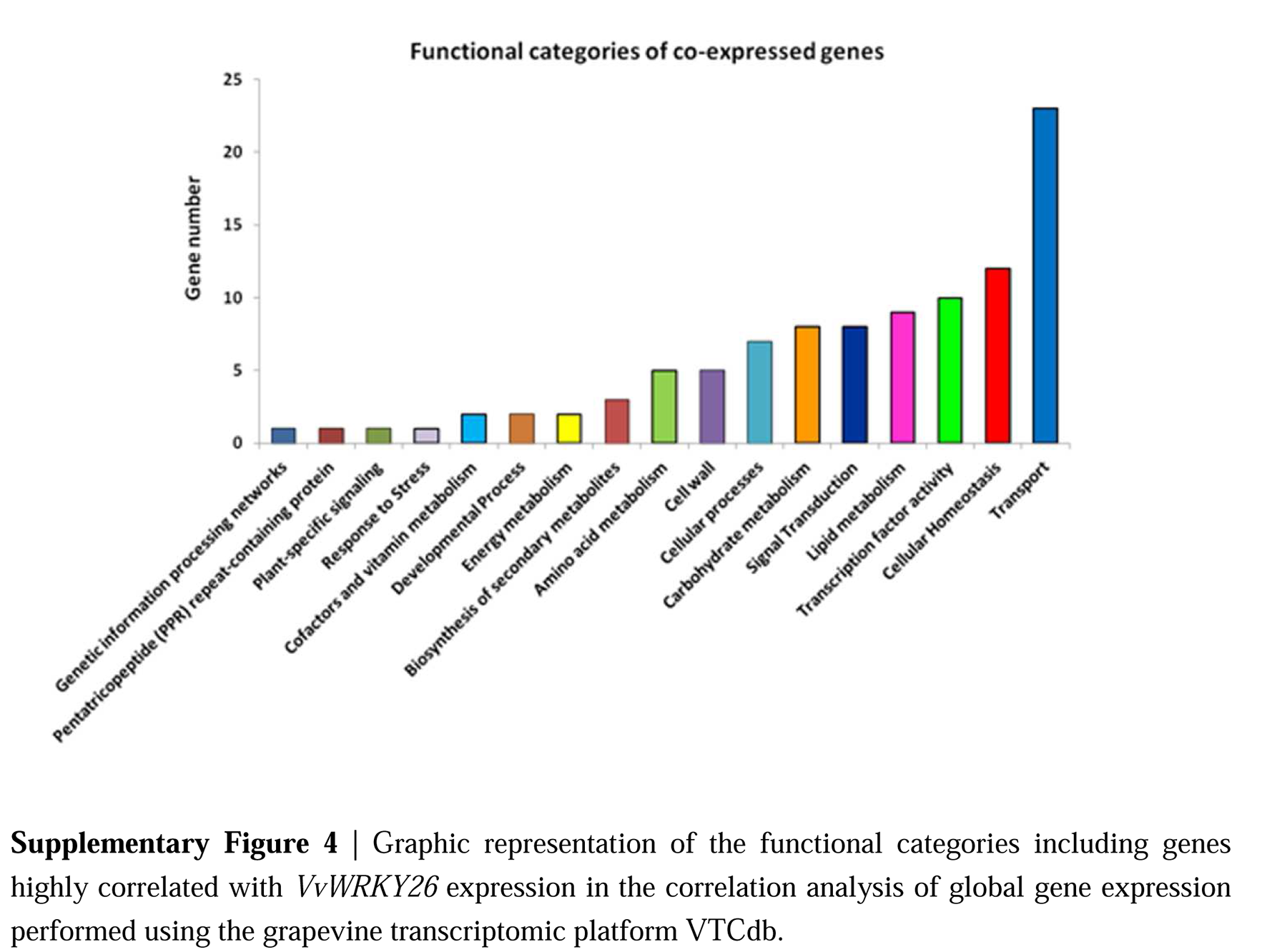

Supplement: Supplementary file 7 [file Image4.TIF]

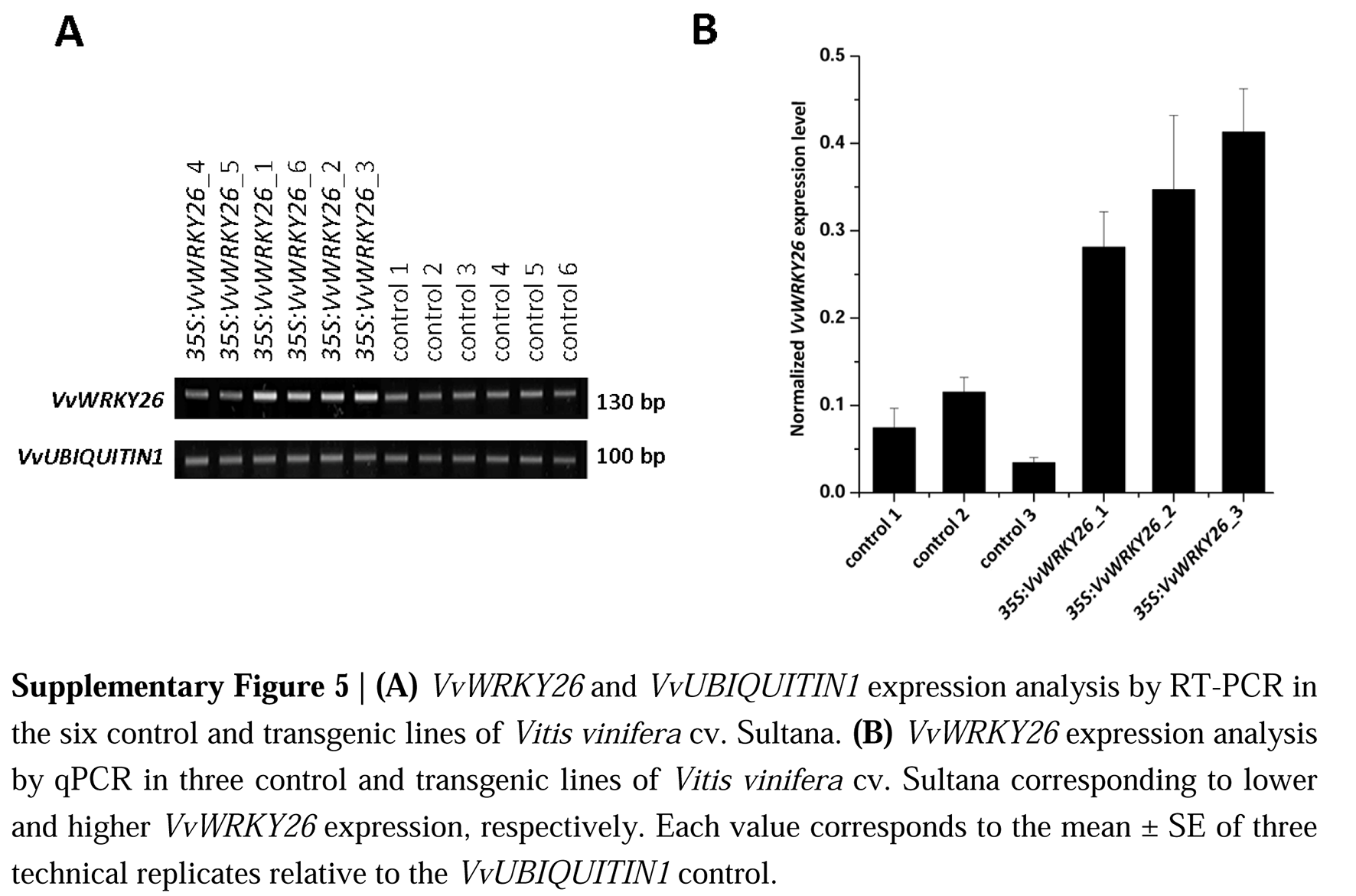

Supplement: Supplementary file 8 [file Image5.TIF]

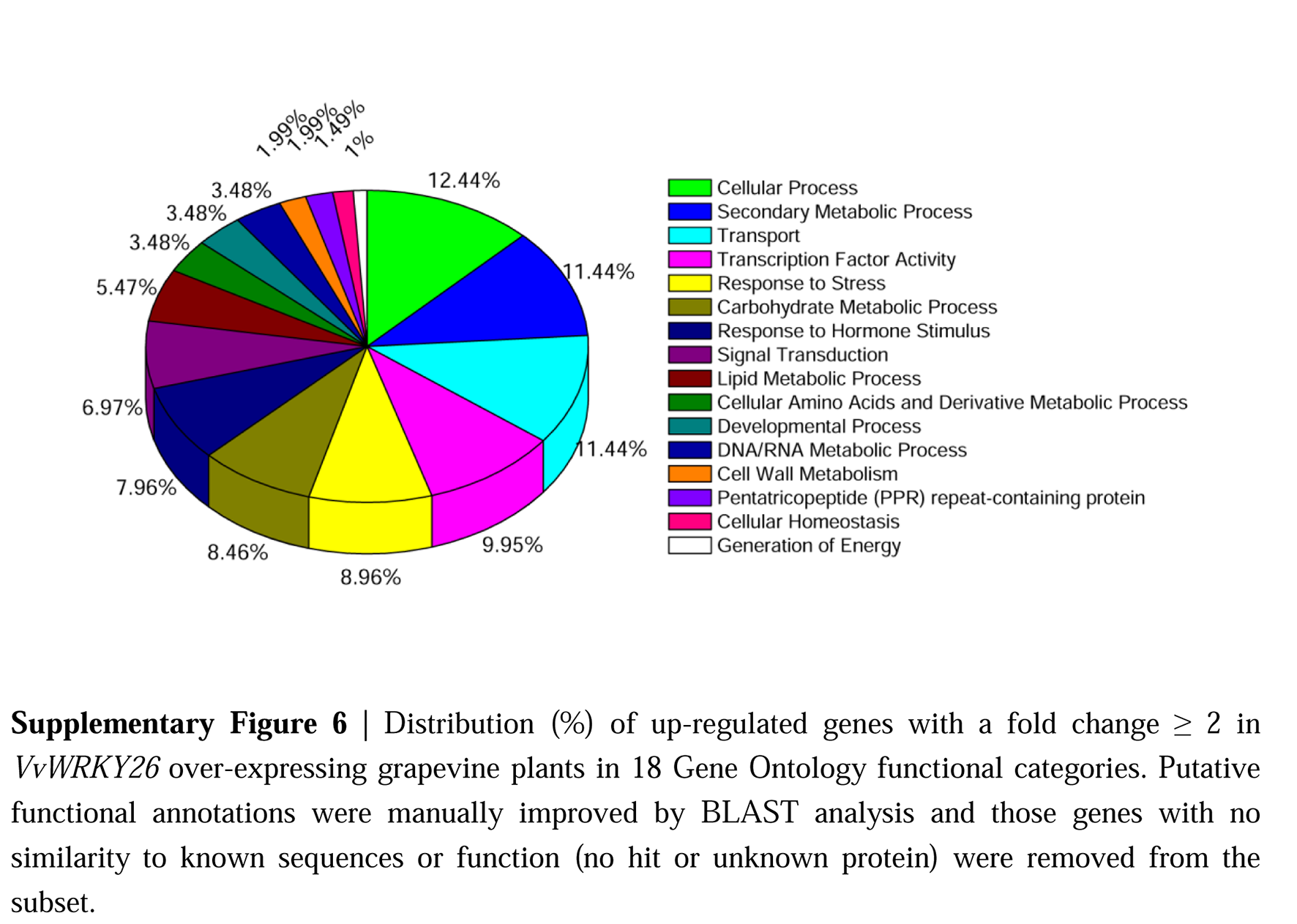

Supplement: Supplementary file 9 [file Image6.TIF]
