# Supplementary material for: A Grapevine TTG2-Like WRKY Transcription Factor Is Involved in Regulating Vacuolar Transport and Flavonoid Biosynthesis
Source: Front Plant Sci. 2017 Jan 5;7:1979. doi: 10.3389/fpls.2016.01979 (PMC5214514; doi:10.3389/fpls.2016.01979)
Supplement: Supplementary file 5 [file Image2.PDF]

```
                                                       D1
VvWRKY26        --------------------------MEIKESERIVVAKPVASRP-TCSSFRSFSELLAG
PhPH3           --------------------------MEVNEAAKIAIARPVASRP-RCPIYRSFSELLAG
AtTTG2          --------------------------MEVNDGERVVIAKPVASRPSSSSGFRTFTELLTD
BnTTG2          --------------------------MEVKESKRVVIAKPVASRPSFTS-IRTFANLLTD
VvWRKY01        -----MAENDETSLSSSASASASRASAPLRPTITLPPRSSMETLFPGGPGFSPGPMTLVS
GhWRKY3         --------------MAAEQGKRSKLSAPTYPTITLPPRPPIDGLFQSGSGLSPGPMTLVS
AtWRKY4         -----------MSEKEEAPSTSKSTGAPSRPTLSLPPRPFSEMFFNGGVGFSPGPMTLVS
VvWRKY33        -----------------MASSAASFNTSSHNSQDDFSFSTQLMSSSFTDLLSSGGDMDHS
PcWRKY1         ------------------------------------------MSSSLGDLLAQPNNDDFG
AtWRKY2         MAGFDENVAVMGEWVPRSPSPGTLFSSAIGEEKSSKRVLERELSLNHGQVIGLEEDTSSN
VpWRKY2         ----------------------------MAGNQSFEAPEAEKAEPNPESDSGSDSDSAES
                                                                           .

                              D2          D1
VvWRKY26        AINASPPTPCPETSFAAIRPKTVRFKPVANRAPIGVVSSQAQVSGTE-------------
PhPH3           AINTSSTNVHSEMGITAIKPKTVRLKPAANYALIGELSSQVGMSGAP-------------
AtTTG2          SVTVSPQTTCHEIVDAAIRPKTLRFNQPVAASVSCPRAEVKGIGNGM-------------
BnTTG2          SVTVPP----HETVDAAIRPKTLRFKQPAAAAASVSCPQVEGNDKGK-------------
VvWRKY01        NFFSDNDPDSYCRSFSQLLAGAMASPAAVPGPRPSFSTDPQVSASSKEDRTSVDAAGDFE
GhWRKY3         AFFSD--PDSTNRSFSQLLAGAMASPGAKLPYNPMDDSFMEVGFEN---------GGEKN
AtWRKY4         NMFPD---SDEFRSFSQLLAGAMSSPATAAAAAAAATASDYQRLGEG----TNSSSGDVD
VvWRKY33        APFAWALSDPLSHTTEFPKFKSLPPPSLPISPPPVSPSSYFAFSS------------GFS
PcWRKY1         S----------NWGFENQKTKSFANQSLPFSPPPVSPSSYFSF-----------------
AtWRKY2         HNKDSSQSNVFRGGLSERIAARAGFNAPRLNTENIRTNTDFSIDSNLRSPCLTISSPGLS
VpWRKY2         GSEDGEGLSDSEGVGAAELGEPRESETLAVASSITESGSQLLSGSALN------------

                                                                         D1
VvWRKY26        --------------------------------------VCNSSDKVLKSDSKPTVVYKPL
PhPH3           --------------------------------------VDCRSDNILQSAEKPKVLYKPM
AtTTG2          --------------------------------------SCDDDS-----DSRNYVVYKPK
BnTTG2          --------------------------------------SCVDS------DTKSYVVYKPK
VvWRKY01        FRFKQNRPS---GLVIAQSP---LFTVPPG--------LSPTCLLDSPGFFSQGP-----
GhWRKY3         SGFKQNRPL---NLGVGNSP---WFTVPPG--------LSPSGLLNSPGLFCLSPQSP--
AtWRKY4         PRFKQNRPT---GLMISQSQSPSMFTVPPG--------LSPAMLLDSPSFLGLFSPVQGS
VvWRKY33        ATDLLDSPLLLSSSNVLPSPTAGNFAAPG---------FNWRSN--SNEHQQAFNDTDRK
PcWRKY1         ----LDSPIQNNNYTIVSSSGNGTMNAQS---------FKEENQNFSDFSFPAQTRPASS
AtWRKY2         PATLLESPVFLSNPLAQPSPTTGKFPFLPGVNGNALSSEKAKDEFFDDIGASFSFHPVSR
VpWRKY2         --------------------------------------------STAQSLASVEFKEQAR

                 D1
VvWRKY26        AKLVSKTTVSLLANLGSSNMSHQQTLAQVEARVQPPNQDRQHSRPHLSSNLHQTFPSQEE
PhPH3           AKLAPRKNISLLENKGSYAPDQKREIAEDEAEGHVQSASEVKQQNGLTTESRQSLLAKSR
AtTTG2          AKLVSKATVSALANMLQGNRQQTWRQSEAVSYGKSVSQGTHRAGPNLVQKVPSFTESE--
BnTTG2          AKLVSQATVSALANMG--NHQQVWRQSEAVPYGKSVSQGTR---PNLVPRVPSFKESE--
VvWRKY01        FVMSHQQALAQVTAQAAQAHSHMQLQAEFPSSLSVSPAASLTQFPSFASNTKAHEQMPPL
GhWRKY3         FGISHQQALAQVTAQAALVQSHVHAQPEYQT---LSAAGSLEPSIPPSSGNPEETSQQML
AtWRKY4         YGMTHQQALAQVTAQAVQANANMQPQTEYPP--------------PSQVQSFSSGQAQIP
VvWRKY33        FSDLLFQSQTRPSGGEALKTQAPWNSDKPEKQTHFPQEKTGVKSEFASLQSLSPEIASIQ
PcWRKY1         TSSSFIPANTNLVEDSLKRKQGGWNFEEPAKKNDFLMDNASVTSDIATLQRISPEMTMNH
AtWRKY2         SSSSFFQGTTEMMSVDYGNYNNRSSSHQSAEEVKPGSENIESSNLYGIETDNQNGQNKTS
VpWRKY2         VCHQEVQTTVTAQTTHVQTKKQLQSSGCPTSSVELSPTSVTQSIQSAPSPTILERRPSPF

VvWRKY26        TDRTSEPSKTASQNLEEDQKPLLPSSNGDRPSYDGYNWRKYGQKQVKGSEYPRSYYKCTY
PhPH3           QDKRIMQSAIVSENTEEVEQSLLNTNNVDRPSYDGYNWRKYGQKQVKGSEYPRSYYKCTH
AtTTG2          ------------------------TSTGDRSSVDGYNWRKYGQKQVKGSECPRSYYKCTH
BnTTG2          ------------------------TSAGDRSSVDGYNWRKYGQKQVKGSDCPRSYYKCTH
VvWRKY01        VSDARTAVKESSGLSQSDQRSQPSSFTVDKPADDGYNWRKYGQKQVKGSEYPRSYYKCTH
GhWRKY3         SSDPQSSAMEYLEASQFDKKSQP-CVAVDKPAEDGYNWRKYGQKQIKGCEYPRSYYKCTH
AtWRKY4         TSAPLPAQRETSDVTIIEHRSQQ-PLNVDKPADDGYNWRKYGQKQVKGSEFPRSYYKCTN
VvWRKY33        TNMQSNNIPQSGRSHHAQPSES---YREQRRSDDGYNWRKYGQKQVKGSENPRSYYKCTF
PcWRKY1         ANMQSNAALQSNLNNYAQSSQSSQTNRDQSKLDDGYNWRKYGQKQVKGSENPRSYYKCTY
AtWRKY2         DVTTNTSLETVDHQEEEEEQRRGDSMAGGAPAEDGYNWRKYGQKLVKGSEYPRSYYKCTN
VpWRKY2         PKANSECMPEGNQKNSSDLKTISTVPSVKTPSADGYNWRKYGQKQVKSPKGSRSYYKCTY
                                                 *********** :*. . .*******

VvWRKY26        PSCPVKKKVERSLDGQIAEIVYKGEHNHSKPQPPKRNSSGTLGQGFVSDGTGQDTNNPAW
PhPH3           LKCPVKKKVERSHDGQIAEIVYRGEHNHPKPQPPKRNFSDGQGRALVSNDTSKETINPAL
AtTTG2          PKCPVKKKVERSVEGQVSEIVYQGEHNHSKPSCPLPRRASSSISSGFQKPPKSIASEGSM
BnTTG2          PKCPVKKKVERSMGGLVSEIVYQGEHNHSKPSCPLPRRASSSSSSGFQRPQRELASEGSI
VvWRKY01        PSCPVKKKVERSLDGQVTEIIYKGQHNHQAPLPNKRAKDTGNPNGNSNFQENPE------
GhWRKY3         PSCPVKKIVERSAEGLITEIIYKSTHNHEKPPPNK--QPKGGSDGNTNSQGNPE------
AtWRKY4         PGCPVKKKVERSLDGQVTEIIYKGQHNHEPPQNTKRGNKDNTANINGSSINNNR------
VvWRKY33        PNCPTKKKVERSLEGHITEIVYKGTHSHPKPQPKRSS-----------------------
PcWRKY1         LNCPTKKKVETTFDGHITEIVYKGNHNHPKPQSTKRS-----------------------
AtWRKY2         PNCQVKKKVERSREGHITEIIYKGAHNHLKPPPNRRSGMQVDGTEQVEQQQQQRDSAATW
VpWRKY2         SDCYAKKIECCDDSGQVIEIIYKSRHNHDPPRKINCMKEGK-------------------
                  * .**       * : **:*:. *.*  *

VvWRKY26        GTRLNERNEGSEGRIENQNEVGLSTHSTYPGKAPLNYDSGTAGALKAGGGTPDNSCGLSG
PhPH3           SNQYPHTREANVHRIENQADVGLSTQTAYCSKPPCFYDP-------TSGAGMYRAFRNSE
AtTTG2          GQDPNNNLYSPLWNNQSNDSTQNRTEKMSEGCVITPFEFAVPRSTNSNPGTSDSGCKSS-
BnTTG2          GQDPSNVYYHPLWSNQSNDSSKSIAEKMNDGCVITPFEFAVPRSANSTGGTSDSGCRSSS
VvWRKY01        ----LASQNQTGNLNKPKEGLPAYSLSKKDQQSSQAIPEHLPGSSDSEEMDDAETRGDEK
GhWRKY3         ----LGSLAVAGNSNNLSEGK----------NHESTQAVELPGFSDCEEGCDEESR-EER
AtWRKY4         ----GSSELGASQFQTNSSNKTKRE--QHEAVSQATTTEHLSEASDGEEVGNGETDVREK
VvWRKY33        -----SQSFPSASTNSEISGHSMPIGNPYMDSMTTSENSSVSIGEDDFDQNSPMSRSGGD
PcWRKY1         -----SSQSYQNSIGTMPE--SSLLENGRSEPVTTPENSSLSFGEDDLFEQGSMNKPGDD
AtWRKY2         VSCNNTQQQGGSNENNVEEGSTRFEYGNQSGSIQAQTGGQYESGDPVVVVDASSTFSNDE
VpWRKY2         ----LSPIGPVTGNSTTADPVRMLNDSDPSTSSKEPVQETPLIPERKRPNSDASDENAEI
```

```
VvWRKY26        DCEEGSK-------------GLEPEEDEPRSKRRKSENQSSETVIVGEGAQEPRIVVQNS
PhPH3           DSAERDK-------------KLEADCDEPKTKRRKIEGQPNGAGTSGE-SALPHMSIQNT
AtTTG2          QCDEGE------------------LDDPSRSKRRKNEKQSSEAG-----------VSQGS
BnTTG2          QCDEGE------------------LDDPSRSKRRKNEKQASQTG-----------VSQSS
VvWRKY01        GEDEP----------------------DPKRR--NTEVRVSDQVSSHRTVTEPRIIVQTT
GhWRKY3         DDDEP----------------------NPKRR--NSTG-EAAVVLSHKAVADAKIIVQTR
AtWRKY4         DENEP----------------------DPKRRSTEVRISEPAPAASHRTVTEPRIIVQTT
VvWRKY33        DENER----------------------EAKRWKGEYEN-EAISASESRTVKEPRVVVQTT
PcWRKY1         DENEP----------------------DSKRWKGEYESNEPMSSLGSRTVREPRIVVQTT
AtWRKY2         DEDDRGTHGSVSLGYDGGGGGGGGEGDESESKRRKLEAFAAEMSGSTRAIREPRVVVQTT
VpWRKY2         KVKEEHID-------------------EPEPKRRTKKSSLGNSGSHFKPGKKPKFVVHAA
                   :                        ..                           :

VvWRKY26        TDSEILGDGFRWRKYGQKVVKGNSYPRSYYRCTSLKCNVRKHVERASEDPGSFITTYEGK
PhPH3           TDSEITEDGFRWRKYGQKVVKGNSYPRSYYRCTSPKCNVRKFVERTIDDPNALITTYEGK
AtTTG2          VESDSLEDGFRWRKYGQKVVGGNAYPRSYYRCTSANCRARKHVERASDDPRAFITTYEGK
BnTTG2          VESDSLEDGFRWRKYGQKVVGGNAHPRSYYRCTSANCRARKHVERASDDPRAFITTYEGK
VvWRKY01        SEVDLLDDGYRWRKYGQKVVKGNPYPRSYYKCTNPGCNVRKHVERAATDPKAVITTYEGK
GhWRKY3         SEVDLLDDGYRWRKYGQKVVKGNPHPRSYYKCTSAGCNVRKHVERASSDPKAVITTYEGK
AtWRKY4         SEVDLLDDGYRWRKYGQKVVKGNPYPRSYYKCTTPGCGVRKHVERAATDPKAVVTTYEGK
VvWRKY33        SDIDILDDGYRWRKYGQKVVKGNPNPRSYYKCTSTGCPVRKHVERSSKDIRAVLTTYEGK
PcWRKY1         SDIDILDDGYRWRKYGQKVVKGNPNPRSYYKCTQVGCPVRKHVERASHDLRAVITTYEGK
AtWRKY2         SDVDILDDGYRWRKYGQKVVKGNPNPRSYYKCTAPGCTVRKHVERASHDLKSVITTYEGK
VpWRKY2         GDVGISGDGYRWRKYGQKMVKGNPHPRNYYRCTSAGCPVRKHIETAIDNTSAVIITYKGI
                 :     **:********:* **. **.**:**   * .**.:* :  :  :.: **:*

VvWRKY26        HNHDMPTRNTNAATSEPDMQAHTNKEKP--------------------------------
PhPH3           HNHGIPSRRPNSEASKTSSKSSAMKDKS--------------------------------
AtTTG2          HNHHLLLSPPSSSTLPFNSPQLSKQTI---------------------------------
BnTTG2          HNHHLNLRPPTSPTLPFTSTQHSNQAI---------------------------------
VvWRKY01        HNHDVPAAKSSSHNTANSIASQKPQ----------------NVVDKKRAFIDNNDQCPIG
GhWRKY3         HNHDVPAARNSSHNTVNNSLPQPKQQ--------------HDAVAEKHSLLQEIDFRNVQ
AtWRKY4         HNHDLPAAKSSSHAAAAAQLRPDNR---------------------PGGLANLNQQQQQQ
VvWRKY33        HNHDVPAARGSGSHFVTKPLPNNSTTTVPAPIRPSVMTNHSNYTTTNANPQTRPPTSASQ
PcWRKY1         HNHDVPAPRGSGSYPAVN-RPSDNTTSAPTAIRP-----TTNYLNPLQNPRAQPAN--GQ
AtWRKY2         HNHDVPAARNSSHGGGGDSGNGNSGGSAAVSHHYHN----GHHSEPPRGRFDRQVTTNNQ
VpWRKY2         HDHDMPVPKKRHGPPSAPLVAVAAPASMNSLQFKKTEAFQNQISSTQWSVDMEGELAAEE
                *:* :

VvWRKY26        ------------------------------------------------------------
PhPH3           ------------------------------------------------------------
AtTTG2          ------------------------------------------------------------
BnTTG2          ------------------------------------------------------------
VvWRKY01        ---RLQLKEEQIT-----------------------------------------------
GhWRKY3         GPAVLRLKEEQIRV----------------------------------------------
AtWRKY4         PVARLRLKEEQTT-----------------------------------------------
VvWRKY33        APFTLEMLQSPGSFGFSGFG---------RMSHPDGVFSRTKE-----------------
PcWRKY1         APFTLEMLQRPRSYEFSGFTNTSNTYAINQNQQASGQFSTAKD-----------------
AtWRKY2         SPFSRPFSFQPHLGPPSGFSFGLGQTGLVNLSMPGLAYGQGKMPGLPHPYMTQPVGMSEA
VpWRKY2         LDPGGEKAMESARTLLSIGFEIKPC-----------------------------------


VvWRKY26        -------------------------------
PhPH3           -------------------------------
AtTTG2          -------------------------------
BnTTG2          -------------------------------
VvWRKY01        -------------------------------
GhWRKY3         -------------------------------
AtWRKY4         -------------------------------
VvWRKY33        ----------EPKDDLFQSFFC---------
PcWRKY1         ----------EPDVDSFFDSFLA--------
AtWRKY2         MMQRGMEPKVEPVSDSGQSVYNQIMSRLPQI
VpWRKY2         -------------------------------
```

Supplementary Figure 2 | Alignment of the Group I WRKY proteins considered in the phylogenetic analysis. The D1 and D2 domains identified by MEME Suite and exclusive of the TTG2 clade are highlighted with squares.
