# Supplementary material for: A Grapevine TTG2-Like WRKY Transcription Factor Is Involved in Regulating Vacuolar Transport and Flavonoid Biosynthesis
Source: Front Plant Sci. 2017 Jan 5;7:1979. doi: 10.3389/fpls.2016.01979 (PMC5214514; doi:10.3389/fpls.2016.01979)
Supplement: Supplementary file 10 [file Table1.PDF]

Supplementary Table 1 | List of primers used.

GENE		SEQUENCE 5'-3'
VvWRKY26	Gene isolation	CACCATGGAGATTAAAGAGTCTGAGA<br>TCATGGTTTCTCTTTATTCGTA
	qPCR	AATGTGCGCAAGCATGTGGA<br>CCTGCATATCTGGTTCAGAG
	ISH probe	TTGAGTAGCTGGAGCCAATG<br>GTCTTAGAGGGCTCGCTTGT
PhF3H	qPCR	TGGAGCTGGACGTAGGATTT<br>CCGGCAATTGTCCACTGACC
PhN21	qPCR	ATGGGAAGCCTTGGTGGTAT<br>GGCTTCTCAACATCGTACTC
PhCHS-A	qPCR	GTGACAGTGGAGGAGTTAT<br>GTTCAGCCACACCTACAA
PhDFR-A	qPCR	AAGCGGACTTGACAGTAG<br>CCTATGGATTTCGAGTCC
PhPH5	qPCR	CCATCTCCAAGACCTGATAG<br>TGCTGTCTGCAAGCCAGTAA
PhPH1	qPCR	CTTGTTCAAAAACCCAGTGGACA<br>TCAGTTCTCGACCCTCCATC
PhACT	qPCR	ATCCCAGTTGCTGACAATAC<br>GGCCCGCCATACTGGTGTGAT
VvPH5	qPCR	CAGGAGGCGTGCAGAAATAG<br>TACCGTATGAGCTGCCTGGA
VvPH1	qPCR	GGGAAGTGTTCTTTCGATCCT<br>TCCCAAGGAATAACGATCTGG
*VvCHS1**	qPCR	AGCCAGTGAAGCAGGTAGCC<br>GTGATCCGGAAGTAGTAAT
*VvMYBPA1**	qPCR	TTGACGGGGTTGACTTCTTC<br>GAGTAGTGATTCGGCGAAGG
*VvUBIQUITIN1**	qPCR	TCTGAGGCTTCGTGGTGGTA<br>AGGCGTGCATAACATTTGCG

*Primer sequences for *VvCHS1, VvMYBPA1* and *VvUBIQUITIN1* are from Goto-Yamamoto et al. (2002), Terrier et al. (2009) and Downey et al. (2003), respectively.
